# Graph-Enhanced Management-Context-Aware Multi-Step Forecasting of Hourly Sensor-Derived Physiological and Behavioral Indicators in Hu Sheep

**DOI:** 10.3390/ani16111670

**Published:** 2026-05-29

**Authors:** Maoxu Wang, Zhixin Gu

**Affiliations:** School of Computer Science and Artificial Intelligence, Northeast Forestry University, Harbin 150040, China

**Keywords:** precision sheep farming, animal-state forecasting, sensor-based monitoring, physiological and behavioral indicators, machine learning

## Abstract

Forecasting sensor-derived animal-state indicators can provide forward-looking information for precision sheep farming, but sheep responses are shaped by their barn environment, previous state, and routine operations. We developed Graph-Enhanced Contextual Long-Range Forecasting for Sheep Farming (GCL-Sheep), a management-context-aware model for multi-step forecasting of active duration, rumination duration, feeding duration, intense exercise duration, and body temperature in Hu sheep. The study used monitoring records from 115 Hu sheep in two farms and three barns, covering monitoring campaigns from March 2024 to December 2025. After domain screening and preprocessing, the data were organized into five farm–season–barn domains, containing approximately 325,000 usable hourly records and 304,000 supervised samples. Barn environmental records, individual physiological/behavioral measurements, and management-operation data were aligned to hourly sequences. GCL-Sheep combines Cross-Variable Graph Construction, hierarchical management-context prefixes, and long-context temporal modeling. For the representative in-domain active-duration forecasting task at the 12 h horizon (H=12), GCL-Sheep reduced the mean absolute error and root-mean-square error by 20.0% and 19.2%, respectively, compared with the second-best baseline, and improved the coefficient of determination by 0.079. In Leave-One-Domain-Out evaluation for active-duration forecasting at H=12, it achieved an average coefficient of determination of 0.792, and few-shot target-domain fine-tuning further improved accuracy. A 96 h historical window achieved the best balance between accuracy and temporal coverage. These results indicate promising retrospective multi-step forecasting performance and suggest that sensor-based animal-state forecasting may provide decision-support information for inspection scheduling and environmental management in sheep farms; however, welfare-threshold-based early-warning and intervention effects still require prospective field validation.

## Simple Summary

Sheep farmers need timely information about animals’ condition, but it is difficult to judge what may happen in the next few hours from barn conditions and electronic monitoring records alone. This study developed a computer-based method to predict five future animal indicators in Hu sheep: time spent moving, time spent chewing cud, time spent feeding, time spent in strong movement, and body temperature. We used records from 115 Hu sheep raised in two farms and three barns in China. The method combined barn environment, previous animal records, and routine farm operations, such as feeding and ventilation. It made more accurate predictions than several commonly used comparison methods. In the main activity prediction test, the main prediction errors were reduced by about one-fifth compared with the best comparison method. The results suggest that electronic monitoring may help farmers plan inspections and adjust barn conditions earlier. However, practical warning rules and the effects of management actions still need to be tested on farms.

## 1. Introduction

In meat sheep production, timely knowledge of physiological and behavioral status is essential for animal welfare assessment, health-risk warning, feeding management, and barn environmental regulation. Precision sheep farming is increasingly supported by wearable sensors, environmental monitoring devices, and automated behavior-recognition systems, which make it possible to continuously record both animal responses and surrounding barn conditions. Recent studies have used triaxial accelerometer-based electronic devices and embedded machine-learning systems for sheep ingestive-behavior discrimination and real-time behavior classification [1,2]. Deep-learning and multimodal signal-fusion methods have also been applied to grazing-sheep behavior classification, spatiotemporal behavior analysis, and fattening Hu sheep behavior recognition [3,4]. Compared with isolated environmental readings such as temperature, humidity, gas concentration, or light intensity, animal-state indicators—including active duration, rumination duration, feeding duration, intense exercise duration, and body temperature—provide an animal-centered information layer that complements barn environmental monitoring. Because these indicators reflect behavioral allocation and physiological state, they are closely related to comfort, stress response, health status, and production management. Specifically, active duration describes general movement and activity allocation; rumination duration and feeding duration characterize ingestive and digestive behavior; intense exercise duration captures abrupt or high-intensity movement events; and body temperature reflects thermoregulatory and physiological status. Previous sheep-monitoring studies have shown that these sensor-derived behavioral and physiological indicators are closely related to feeding management, health-risk warning, welfare assessment, and environmental adaptation [1,4,5,6,7,8]. These indicators therefore form a practical information layer between field sensing and farm decision-making, and they are suitable targets for artificial intelligence models designed to support animal-state forecasting and management-oriented decision support in smart sheep farming.

However, farm managers do not only need to know the current state of their animals; they also need forward-looking information that can create a time window for intervention. Forecasting sheep physiological and behavioral indicators is challenging because their future trajectories may be influenced by short-term environmental exposure, previous animal-state dynamics, and routine management events, including feeding, manure cleaning, ventilation adjustment, equipment operation, and day–night rhythms [8,9,10]. Previous studies have shown that environmental stressors, such as temperature–humidity variation and air-quality deterioration, can affect feeding behavior, activity patterns, thermoregulatory responses, and other physiological or behavioral states in sheep [6,7]. These inputs differ in sampling frequency, biological meaning, and operational context, while animal responses may involve cross-variable associations and delayed effects. Therefore, multi-step forecasting requires the joint modeling of environmental exposure, historical animal-state information, and management context, rather than relying on isolated sensor readings. Accurate multi-step forecasting can therefore help identify transitions from comfort to stress and from normal to abnormal status before they become evident in routine observation, supporting health early warning, anomaly detection, precision feeding, inspection scheduling, and environmental regulation in sheep farming [11,12].

Previous livestock monitoring studies have demonstrated the value of statistical and machine-learning models for converting farm data into production or welfare information. Statistical forecasting pipelines established the basis for short-term sequence prediction [13,14], and livestock applications used environmental and age variables to predict thermal responses [15]. Regression and machine-learning methods have also been used to predict ammonia exposure risk, ammonia concentration, and barn temperature in livestock and poultry production [16,17,18]. These approaches remain useful because of their interpretability and relatively low deployment cost, but they often depend on manually selected windows and engineered features. For continuous sheep monitoring, this limits their ability to represent cross-scale temporal dependencies, non-stationary animal responses, and interactions among environmental, physiological, behavioral, and management-related variables [19,20].

Recent studies in precision sheep farming have increasingly used sensor and time-series data to characterize animal behavior and welfare-related states. Sheep-specific studies have applied triaxial accelerometer-based electronic devices and embedded machine-learning systems to ingestive-behavior discrimination and real-time behavior classification [1,2]. Deep-learning and multimodal signal-fusion methods have also been used for grazing-sheep behavior classification, spatiotemporal behavior analysis, and fattening Hu sheep behavior recognition [3,4]. Related work has also explored multimodal welfare-indicator prediction in dairy sheep [21]. However, these sheep-specific studies have mainly focused on current-state monitoring, behavior recognition, or welfare-related indicator characterization, whereas direct studies on multi-step individual-level forecasting of future physiological and behavioral indicators in meat sheep remain limited. In particular, few studies have examined how to forecast active duration, rumination duration, feeding duration, intense exercise duration, and body temperature under farm–season–barn distribution shifts [8,10]. Because directly comparable sheep-specific forecasting studies are still scarce, representative forecasting studies in other livestock production systems are considered only as methodological references, including sequential prediction of environmental variables in pig houses, lightweight prediction of NH_3_ concentration, activity-based prediction of reproductive risk in dairy cows, and generative modeling for livestock behavior forecasting and missing-data imputation [22,23,24,25]. Taken together, these studies highlight the precise gap addressed in this work: multi-step, individual-level forecasting of sensor-derived physiological and behavioral indicators in meat sheep under domain shifts, using barn environmental exposure, historical animal-state information, and management-context data jointly.

A key difficulty in this task is that meat sheep responses should not be interpreted from environmental variation alone. Routine management and temporal context, such as feeding stage, manure cleaning, ventilation status, equipment operation, and day–night rhythm, may influence observed activity patterns and the interpretation of similar numerical fluctuations [8,9,10]. Previous sheep studies have also shown that feeding-related behavior, activity patterns, and physiological responses are associated with sensor-derived behavioral information and environmental stress conditions [1,4,6,7]. For example, an increase in activity may correspond to normal post-feeding movement under one management context, whereas a similar numerical change under unfavorable thermal or barn-operational conditions may require a different welfare-oriented interpretation. Therefore, sheep-state forecasting should incorporate management context together with environmental and animal-state histories. Existing decision-support studies in precision sheep farming have emphasized that sensor readings should be interpreted together with operational background and expert knowledge, rather than as isolated values [9,21]. For example, increased activity may indicate a normal post-feeding response or an abnormal reaction to environmental deterioration, and elevated body temperature may reflect short-term activity or a delayed physiological response after sustained thermal load. Therefore, sheep-state forecasting should be treated as a management-context-aware precision livestock problem: the model must learn variable relationships, use farm operation information, and capture long-range cumulative and lagged responses within the same forecasting process [26,27].

To address this applied gap, this study proposes GCL-Sheep (Graph-Enhanced Contextual Long-Range Forecasting for Sheep Farming), an AI model for forecasting key physiological and behavioral indicators in meat sheep from multi-source farm monitoring data. The model supports multi-step prediction of active duration, rumination duration, feeding duration, intense exercise duration, and body temperature through target-specific forecasting tasks under a unified modeling strategy [28]. GCL-Sheep uses a Cross-Variable Graph Construction (CGC) module to represent coupling between barn environmental variables and animal-state indicators, a hierarchical Domain Knowledge Prefix Encoding module (hierarchical DKPE) to include farm, season, barn, and management-event context, and a Long-Context Prediction Backbone (LCPB) to model cumulative exposure and lagged animal responses. Based on multi-scenario data organization, the model is evaluated under in-domain forecasting, Leave-One-Domain-Out cross-validation (LODO), and few-shot adaptation, so that its accuracy, robustness, and potential for deployment in new farm scenarios can be assessed [29,30].

The main contributions of this study are as follows:

(1) This study formulates multi-step individual-level forecasting of sensor-derived physiological and behavioral indicators in Hu sheep under farm–season–barn distribution shifts as a precision sheep farming task.

(2) This study develops GCL-Sheep, which combines domain-informed graph structure, data-driven variable association learning, hierarchical management-context encoding, and long-context temporal modeling. Compared with conventional graph-based or Transformer-based forecasting models, the proposed model explicitly integrates environmental exposure, animal-state history, and farm operation context within one forecasting framework.

(3) This study evaluates GCL-Sheep under in-domain forecasting, Leave-One-Domain-Out cross-domain testing, few-shot target-domain adaptation, ablation analysis, and sensitivity analysis, providing retrospective evidence for management-context-aware animal-state forecasting in precision sheep farming.

## 2. Materials, Data Collection, and Preprocessing

This section describes the farm monitoring data and preprocessing workflow used to construct forecasting samples for precision sheep farming. The data include barn environmental measurements, individual physiological and behavioral records of meat sheep, and management-context information. Because these data sources differ in sampling frequency, scale, noise level, and biological meaning, preprocessing was performed to organize farm scenarios, align temporal resolution, handle missing values and local anomalies, standardize features, and generate supervised multi-step forecasting samples while preserving the relationship between environmental exposure, animal state, and management operations.

### 2.1. Multi-Scenario Data Acquisition and Collection Settings

The data collection in this study covered two typical meat sheep farming environments in China, namely, Daqing in Heilongjiang Province, located in the cold northeastern region, and Aksu in Xinjiang, located in the arid northwestern region. These two sites differ substantially in climatic background, seasonal variation, barn operating conditions, and environmental control strategies, thereby providing natural distribution-shift conditions for cross-scenario modeling.

The Daqing experimental site was located at Woyuan Agriculture in Zhaozhou County, Heilongjiang Province. This farm contains 20 large-scale fattening sheep barns with a total area of 3624.31 m^2^, and the resting area inside each barn is equipped with bamboo slatted flooring with 13 cm spacing. In this study, two standard barns, each measuring 30 m in length and 5 m in width, were selected as monitoring targets. Each barn housed 40 Hu sheep with body weights ranging from 15 to 55 kg. This region is characterized by hot, rainy summers and extremely cold winters, with winter minimum temperatures falling below −20 °C. As a result, the thermal insulation demand is high and ventilation is substantially restricted, leading to strong seasonal variation in environmental exposure conditions.

The Aksu experimental site was located at Xincheng Agriculture in Aksu, Xinjiang, on the edge of the Tarim Basin, which is a typical warm temperate arid climatic zone. The experimental barn was a standard enclosed sightseeing sheep barn measuring 70 m in length and 9 m in width, with an overall fattening capacity of approximately 150 sheep. For individual-state monitoring, 35 Hu sheep equipped with commercial intelligent electronic ear tags were selected as key observation subjects, with an initial average body weight of 45.0 ± 2.5 kg. The electronic ear-tag system used in this study was a commercial livestock-monitoring device supplied by Zhongke Muyun Intelligent Livestock Technology Co., Ltd., (Xi’an, China), model MY-ET-S01. Each ear tag integrated an individual identification module, a triaxial acceleration sensing module, a temperature sensing module, and a wireless data-transmission module. The individual identification information was recorded through the ear-tag ID and two-dimensional code, while the triaxial acceleration and temperature signals were used by the device platform to derive individual animal-state variables, including active duration, rumination duration, feeding duration, intense exercise duration, and body temperature.

The raw motion and temperature signals were collected automatically by the ear-tag device at a nominal sensing interval of 5 min and were uploaded wirelessly to the barn-level receiving terminal through a LoRa-based communication module. The receiving terminal then transmitted the records to the cloud-based data management platform through a 4G network. According to the manufacturer’s specifications, the temperature record had a resolution of 0.1 °C and a nominal measurement accuracy of ±0.3 °C within the common physiological temperature range of sheep. The device platform exported individual-level records at an hourly temporal resolution, which were subsequently aligned with barn environmental data and management-context data. Before deployment, all ear tags were checked for ID consistency, attachment status, data-upload stability, clock synchronization, and abnormal or missing records.

**Figure 1:**
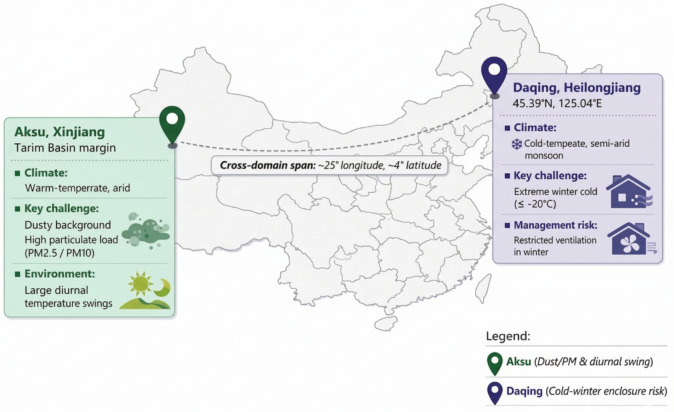
Geographic distribution of the experimental sites.

This scenario is characterized by high dust exposure, pronounced diurnal temperature variations, and low air humidity. The barn was equipped with a 30 kW hot-air blower and exhaust devices to coordinate heating and ventilation demands. As shown in Figure 1, the Daqing and Aksu experimental sites are located in the northeastern and northwestern parts of China, respectively, spanning approximately 25 degrees of longitude and 4 degrees of latitude.

To clarify the comparability of the monitored animals and farm-management conditions across sites, all monitored animals were Hu sheep raised under routine commercial fattening management. The monitored animals in both regions belonged to the fattening production stage, although the body-weight range differed between scenarios because of farm-specific production batches. During the selected monitoring periods, no large-scale disease outbreak or major abnormal veterinary intervention was recorded in the monitored barns, and records with abnormal or incomplete animal-state sequences were excluded during data-quality screening.

The two farms followed routine feeding and daily management schedules for commercial fattening sheep, but the exact diet formulation, stocking density, and environmental control operations were not forced to be identical, because the purpose of this study was to evaluate forecasting under real farm–season–barn distribution shifts. Based on barn floor area and housed capacity, the approximate space allowance was about 3.75 m^2^ per sheep in the monitored Daqing barns and about 4.20 m^2^ per sheep in the Aksu barn at its nominal fattening capacity. Therefore, breed, production purpose, monitored variable definitions, and data-collection modalities were kept comparable across sites, whereas differences in local management practice, stocking condition, diet formulation, and environmental control strategy were treated as part of the domain differences to be modeled. Time-varying management-context variables, including feeding or fasting stage, manure cleaning, vaccination, and heater, fan, or exhaust-device status, were recorded to help represent these operational differences during forecasting.

**Figure 2:**
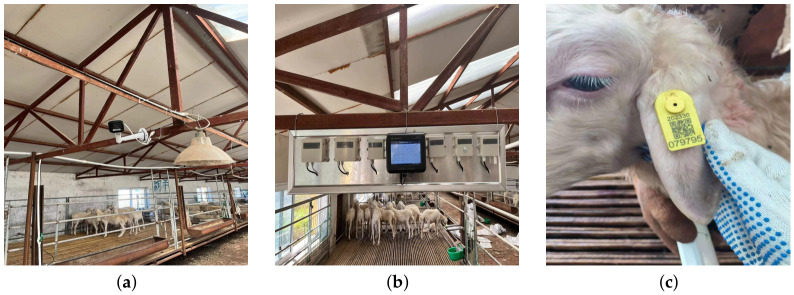
Representative photographs of the on-farm monitoring setup. The panels show (**a**) barn environment and pen arrangement, (**b**) environmental monitoring system used for barn environmental data collection, and (**c**) electronic ear tag used for individual sheep identification and animal-state monitoring.

To improve the transparency of the experimental environment and monitoring configuration, representative photographs of the on-farm monitoring setup are provided in Figure 2. These photographs show the real barn environment and pen arrangement, the installed environmental monitoring system, and the electronic ear tag used for individual sheep identification and animal-state recording. These visual materials are provided to help readers understand the animal arrangement, sensor deployment, and practical monitoring conditions used in this study.

Both experimental scenarios synchronously collected three types of data: 

(1) Environmental monitoring data, including temperature, relative humidity, NH_3_, CO_2_, PM_2.5_, PM_10_, and light intensity;

(2) Individual physiological and behavioral data, including active duration, rumination duration, feeding duration, intense exercise duration, and body temperature;

(3) Management-context data, including day–night status, feeding/fasting stage, manure cleaning operations, vaccination, and the on/off status of heaters, fans, and exhaust equipment. The two scenarios were kept consistent in terms of monitoring modalities and variable definitions, thereby providing a basis for subsequent unified modeling. The monitored variables were selected to cover three complementary dimensions of precision sheep farming: barn environmental exposure, individual animal-state responses, and management-context information. This design was intended to support the joint interpretation of environmental conditions, physiological and behavioral responses, and routine production operations, rather than treating each sensor reading as an isolated numerical input. The biological, environmental, and practical management relevance of the monitored variables is summarized in Table 1. The selection of these variables was also supported by previous studies on sheep monitoring, sensor-based behavior recognition, and environmental stress responses.

At the raw sampling level, environmental monitoring data were collected continuously at the minute scale, with original sampling intervals ranging from 1 to 5 min across scenarios. Across the original individual-level monitoring records, approximately 470,000 individual hourly records were obtained before domain screening. Among them, the five final modeling domains D1–D5 contained approximately 349,000 raw individual hourly records, whereas approximately 121,000 records from the summer and autumn portions of Dataset B were not assigned to independent modeling domains because of production-batch transition, monitoring-system adjustment, and insufficient continuity of synchronized individual ear-tag records. Environmental records and management-context records were aligned to the same hourly time index during preprocessing.

To improve reproducibility, we report the approximate number of records retained after domain screening and each preprocessing step in Table 2. Because the final forecasting samples were generated at the individual-hour level, the table summarizes individual hourly records after quality control, missing-value processing, temporal alignment, and sliding-window generation. The supervised sample counts were calculated under the default historical lookback window L=96 and the maximum forecasting horizon H=12; shorter forecasting horizons generated slightly more candidate windows.

### 2.2. Multi-Scenario Data Organization and Domain Construction

The integrated multi-scenario dataset consists of three original datasets, corresponding to the Daqing summer scenario, the Daqing cross-season long-term scenario, and the Aksu autumn–winter scenario, respectively. Specifically, Dataset A was collected from Barn #1 in Daqing, Heilongjiang, during June 2024–October 2024; Dataset B was collected from Barn #2 in Daqing, Heilongjiang, during March 2024–June 2025; and Dataset C was collected from Barn #3 in Aksu, Xinjiang, during September 2025–December 2025. These three datasets differ substantially in terms of geographic region, seasonal conditions, barn environmental background, and management context.

To explicitly represent distributional differences across scenarios, we organize the data into a set of domains according to the triplet farm–season–barn. A single domain is defined as follows:

(1) d=(f,s,b)

where f denotes the farm or experimental site, s denotes the seasonal condition, and b denotes the barn identifier or barn source. Under this organization scheme, samples from different seasons within the same region, different barns within the same region, and different geographic regions can all be treated as independent but structurally consistent time-series domains. This data organization supports both in-domain time-series forecasting and cross-domain generalization evaluation.

After data quality screening and seasonal segmentation, the three original datasets were reorganized into five farm–season–barn domains. Although Dataset B covered a long monitoring period spanning four calendar seasons, only the winter and spring segments of Dataset B were used as independent modeling domains. The summer and autumn portions of Dataset B were not assigned to additional domains because they overlapped with production-batch transition, temporary adjustment of the monitoring system, and changes in the set of continuously tracked individual ear-tag IDs. These factors reduced the continuity of synchronized individual-state records, environmental records, and management-context logs after quality control and temporal alignment. Therefore, these segments did not satisfy the stable-cohort and continuous time-series requirements for constructing independent domain-level forecasting samples. In addition, the Daqing summer condition was already represented by Dataset A. The final modeling domains were therefore restricted to five quality-controlled farm–season–barn units.

Specifically, D1 represented the Daqing summer Barn #1 scenario from Dataset A; D2 and D3 represented the Daqing winter and spring Barn #2 scenarios from Dataset B, respectively; and D4 and D5 represented the Aksu autumn and winter Barn #3 scenarios from Dataset C, respectively. Thus, the domain definition used in this study was based on quality-controlled seasonal farm–barn units rather than raw datasets. The five domains D1–D5 were used for subsequent in-domain forecasting, Leave-One-Domain-Out cross-domain evaluation, and few-shot adaptation analysis. The detailed division of the original data sources and domain assignment is shown in Table 3.

### 2.3. Temporal Alignment and Resampling

Because environmental monitoring data are typically collected continuously at the minute level, whereas individual physiological and behavioral data are mainly recorded at the hourly level, the two data types are not consistent in temporal resolution. To ensure a unified time scale while minimizing information leakage caused by frequency conversion, we do not upsample the hourly individual-level data. Instead, we use the hour as the unified temporal granularity and aggregate high-frequency environmental data into hourly features within fixed time windows.

For any environmental variable x, its mean value within the t-th hourly window is defined as follows:

(2) $${\bar{x}}_{t}=\frac{1}{n_{t}}\sum_{i=1}^{n_{t}}x_{t,i}$$

where $n_{t}$ denotes the number of observations within the t-th time window, and $x_{t,i}$ denotes the i-th observation in that window. Considering that the mean alone is insufficient to fully capture environmental fluctuations and short-term disturbance characteristics, we further extract additional statistics, including the maximum, minimum, standard deviation, and end-of-window value, which together constitute the environmental feature vector for the current hour, denoted as $x_{t}^{\mathrm{env}}$. The individual physiological/behavioral data and management-context data are also aligned to hourly time steps using the same temporal index, so as to ensure that environmental features, individual-state features, and contextual features can be matched on a unified time scale.

### 2.4. Missing-Value Processing and Feature Standardization

Due to sensor noise, network latency, equipment instability, and temporary communication interruptions, missing values and local anomalies were inevitable in the raw time-series data. To make the preprocessing procedure more transparent, the approximate missingness rate of the main variables before imputation or window removal is summarized in Table 4. Since the final modeling samples were organized as farm–season–barn domains, the missingness statistics are reported at the domain level rather than only at the raw dataset level.

Overall, the missingness rate was relatively low for most variables. Temperature, relative humidity, light intensity, and management-context variables generally had missingness rates below 2%. Gas and particulate variables showed slightly higher missingness, especially in the Aksu domains, mainly because of dust exposure and temporary sensor or communication instability. The individual physiological and behavioral indicators derived from electronic ear tags also showed low-to-moderate missingness, mainly caused by temporary data-upload failure, loose attachment, or short communication interruptions. These statistics were used to determine whether a missing segment could be safely interpolated or whether the corresponding time window should be removed.

Different types of data were processed separately before model training. For short-term missing values in the environmental data, linear interpolation was used when the length of consecutive missing observations did not exceed two sampling points. When the consecutive missing length exceeded two sampling points but did not exceed 1 h, forward filling was applied only to preserve local continuity within the same hourly window. If the missing duration exceeded 1 h, the corresponding time window was removed. For the individual physiological/behavioral data, samples with incomplete or abnormal animal-state sequences were screened according to missing length and data continuity, so as to minimize the impact of device-related errors on supervised forecasting samples. For the management-context data, missing values were preferentially recovered based on log records, equipment states, or time-based rules; if reliable recovery was not possible, the state was marked as unknown. Because the missingness rates were generally low and long missing segments were removed rather than filled, interpolation and forward filling were used only as local corrections and were not expected to substantially alter the overall time-series structure.

In addition, because different variables vary substantially in scale and value range, directly feeding them into the model may cause the training process to be biased toward large-scale variables. To address this issue, continuous features were standardized using statistics estimated only from the corresponding training data of each experimental split. For any feature $x_{t}$, its standardized form is defined as follows:

(3) $$x_{t}^{*}=\frac{x_{t}-\mu_{\mathrm{train}}}{\sigma_{\mathrm{train}}}$$

where $\mu_{\mathrm{train}}$ and $\sigma_{\mathrm{train}}$ denote the mean and standard deviation estimated from the training set, respectively. In the in-domain setting, these statistics were computed only from the chronological training subset within the corresponding domain and were then applied to the validation and test subsets of the same domain. In the Leave-One-Domain-Out setting, the standardization parameters were computed only from the merged source-domain training data and were then applied to the source-domain validation data and the held-out target-domain test data. The held-out target domain was never used to estimate standardization parameters. In the few-shot adaptation setting, the same source-training standardization parameters were retained, and the target-domain adaptation samples were used only for model adaptation rather than for re-estimating normalization statistics. This procedure avoids data leakage and ensures that cross-domain performance reflects generalization to unseen farm–season–barn conditions.

### 2.5. Supervised Sample Generation

For the multivariate time-series forecasting task considered in this study, the model input is not a static feature vector at a single time point, but a temporal window composed of multiple consecutive historical time steps. Therefore, instead of constructing additional lag features separately, we directly organize historical sequence samples using a sliding-window strategy, enabling the model to learn the temporal dependency relationships among environmental variables, individual physiological and behavioral indicators, and management context within a unified input. After temporal alignment, missing-value processing, and feature standardization, the environmental features, individual physiological/behavioral features, and management context features at each hour are concatenated to form a joint feature vector at time step t, denoted as $z_{t}$. On this basis, supervised learning samples are generated using a sliding-window scheme. Let *L* denote the lookback window length and *H* denote the forecasting horizon. Then, the input sample at time step *t* is defined as follows:

(4) $$X_{t}=\left[z_{t-L+1},z_{t-L+2},\ldots ,z_{t}\right]$$

The five monitored physiological and behavioral indicators were treated as target-specific forecasting tasks under a unified modeling framework, rather than as a single multi-output joint forecasting task. Let Q={Act,Rum,Feed,IntEx,BT} denote the set of target indicators, corresponding to active duration, rumination duration, feeding duration, intense exercise duration, and body temperature, respectively. For each target indicator q∈Q, a separate supervised target sequence is constructed as follows:

(5) $$Y_{t}^{\left(q\right)}=\left[y_{t+1}^{\left(q\right)},y_{t+2}^{\left(q\right)},\ldots ,y_{t+H}^{\left(q\right)}\right],\;q\in Q$$

where $Y_{t}^{\left(q\right)}$ denotes the future *H*-step sequence of the target indicator *q*. The same input feature window $X_{t}$, containing barn environmental features, historical animal-state indicators, and management-context variables, is used for all target-specific tasks. During model training and evaluation, each indicator is forecasted with a target-specific prediction head under the same GCL-Sheep architecture and experimental protocol. This design allows the model to use the full multi-source historical context while reporting forecasting performance separately for each physiological or behavioral indicator.

Through the above procedure, the raw multi-source heterogeneous monitoring data are uniformly transformed into supervised sample sets suitable for subsequent time-series forecasting. The organization of the preprocessed single-time-step data is illustrated in Table 5.

Each row in the table corresponds to the single-time-step features of one meat sheep at a given hourly time step. The environmental features are used to describe the statistical characteristics of each environmental variable within the current hourly window, mainly including the mean, maximum, minimum, standard deviation, and end-of-window value of ambient temperature, relative humidity, ammonia concentration, carbon dioxide concentration, PM_2.5_, PM_10_, and light intensity. The physiological/behavioral features represent the individual state at that time step, mainly including active duration, rumination duration, feeding duration, intense exercise duration, and body temperature. The management-context features correspond to discrete state information related to farming operations, mainly including temporal rhythms, operational activities, and environmental control states. Because the actual feature dimensionality is relatively high, only a subset of representative fields is listed in the table, while the remaining variables are omitted and indicated by ellipses. At the model input stage, multiple consecutive time steps are further organized into historical sequence samples using a sliding-window strategy. For each target-specific task, one physiological or behavioral indicator over the subsequent future time steps is used as the prediction output, while the full multi-source historical feature window is retained as the model input.

### 2.6. Data Splitting and Evaluation Settings

After supervised sample construction, the subsequent experiments were organized from two perspectives—namely, in-domain forecasting and cross-domain forecasting—so as to simultaneously evaluate the model’s fitting ability under the same distribution and its generalization ability in unseen scenarios. Let the integrated set of multi-scenario domains be denoted as

(6) $$D=\{d_{1},d_{2},\ldots ,d_{M}\}$$

where each domain $d_{m}$ is jointly defined by farm, season, and barn, i.e.,

(7) $$d_{m}=\left(f,s,b\right)$$

Under this organization scheme, all subsequent experiments were conducted on unified hourly sequences and a unified sliding-window sample representation.

For the in-domain forecasting setting, the samples within each domain were divided into training, validation, and test sets in chronological order, at a ratio of 7:1:2. To avoid temporal leakage, the splitting process strictly preserved the original temporal order, and no random shuffling was applied. Supervised learning windows were generated independently within each subset and did not cross the boundaries between the training, validation, and test sets. This setting was mainly used to evaluate the model’s ability to fit the variation trends of target indicators under the same farm–season–barn condition, and it was kept consistent with the subsequent multi-step forecasting experiments, lookback-window experiments, and ablation studies.

For the cross-domain forecasting setting, we adopted the Leave-One-Domain-Out (LODO) protocol. Specifically, in the *m*-th experimental round, the domain $d_{m}$ was selected as the target-domain test set, while the remaining M−1 domains were merged to form the source-domain sample set:

(8) $$D_{m}^{src}=D\setminus \left\{d_{m}\right\}$$

The model was trained and validated using only the source-domain data, without using any target-domain samples for parameter updating. After training, the model was directly applied to the target domain for testing, so as to evaluate its transferability under unseen farms, unseen seasons, or unseen barns. This setting provides a stricter test of whether the model has learned transferable environment–animal-state association patterns across scenarios, rather than merely relying on local statistical patterns from a single scenario.

During source-domain training, to ensure the stability of model selection and early stopping, the source-domain samples were further split into training and validation sets in chronological order. The validation set was used for early stopping, parameter selection, and hyperparameter tuning. For target-domain testing, all samples remained completely independent and were used only for final performance evaluation. Unless otherwise specified, the subsequent cross-domain experiments report the average results over all LODO rounds, so as to reduce the randomness caused by any single target-domain split.

In addition, to simulate the practical situation in which only a small amount of target-domain observational data is available during the early deployment stage of a new scenario, we further introduced a few-shot target-domain fine-tuning setting in addition to zero-shot cross-domain testing. For each LODO round, the model was first trained using only the source-domain data, without using any target-domain samples. Then, the chronologically earliest 5%, 10%, or 20% of the held-out target-domain supervised samples were used as the adaptation set. The remaining later samples from the same target domain were used only for testing. The adaptation samples were strictly excluded from the test set, and supervised windows were generated independently within the adaptation and test subsets so that no sliding window crossed the adaptation–test boundary.

During few-shot target-domain fine-tuning, the model was initialized with the source-domain trained parameters from the corresponding zero-shot LODO round and then updated on the target-domain adaptation set using a smaller learning rate and a limited number of fine-tuning epochs. All trainable parameters of GCL-Sheep, including the data-driven graph parameters, hierarchical context-encoding parameters, long-context backbone, and prediction head, were updated during fine-tuning, whereas the predefined static prior graph topology was kept unchanged. The held-out target-domain test samples were not used for parameter updating, stopping, or model selection. Because fine-tuning started from the trained source-domain model and used only 5%, 10%, or 20% of the target-domain samples, the computational cost was much lower than that of training a new model from scratch.

Through the above three settings—namely, in-domain forecasting, zero-shot cross-domain forecasting, and few-shot target-domain fine-tuning—this study systematically evaluated the predictive accuracy, cross-scenario transferability, and limited-data adaptation ability of GCL-Sheep for forecasting key physiological and behavioral indicators in meat sheep.

### 2.7. Rationale for Preprocessing and Experimental Settings

The main preprocessing and experimental settings were determined by combining the characteristics of the monitoring devices, time-series forecasting requirements, preliminary data-quality inspection, and the biological and practical relevance of the monitored variables. First, an hourly temporal granularity was adopted because the individual physiological and behavioral records exported by the electronic ear-tag platform were organized as hourly records, whereas the environmental variables were originally collected at a higher frequency. Aggregating environmental measurements to hourly statistics avoided artificial upsampling of individual animal-state records and provided a unified temporal scale for environmental exposure, animal response, and management-context information.

Second, the missing-value processing rules were designed according to the raw sampling frequency and the continuity of environmental changes. Short gaps in environmental records were regarded mainly as temporary sensor or communication interruptions and were corrected by linear interpolation when no more than two consecutive observations were missing. For gaps longer than two sampling points but not exceeding 1 h, forward filling was used only to preserve local continuity within the same hourly window. Time windows with missing durations longer than 1 h were removed because they could no longer reliably represent the corresponding hourly environmental exposure. For individual physiological and behavioral records, samples with incomplete or abnormal animal-state sequences were screened to avoid propagating device-related errors into the supervised forecasting samples.

Third, the temporal-window settings were selected to balance biological interpretability, forecasting demand, and computational cost. The forecasting horizons of 1, 3, 6, and 12 h were used to represent short-term and medium-term early-warning intervals that are relevant for barn inspection, ventilation adjustment, feeding management, and targeted intervention. The default lookback window was set to 96 h after preliminary validation and sensitivity analysis, because this range covers multiple day–night cycles and provides sufficient historical context for delayed animal responses without introducing excessive redundant information.

Fourth, the chronological data-splitting strategy was adopted to avoid temporal leakage. In the in-domain setting, training, validation, and test sets were separated according to temporal order, and sliding windows were generated independently within each subset. In the cross-domain setting, the Leave-One-Domain-Out protocol was used to evaluate whether the model could generalize to unseen farm–season–barn conditions. These settings were chosen to better approximate practical deployment, where future samples and new farming scenarios are not available during model training.

Finally, the monitored physiological and behavioral variables were selected according to their availability from the ear-tag platform and their relevance to sheep welfare and production management. Active duration, rumination duration, feeding duration, intense exercise duration, and body temperature provide complementary information on activity allocation, ingestive behavior, abnormal movement, and thermoregulatory status. Previous studies on sheep monitoring, sensor-based behavior recognition, and environmental stress responses have also shown that these types of behavioral and physiological indicators are closely related to welfare assessment, feeding behavior, health status, and environmental adaptation [1,2,4,6,7,8,10].

## 3. GCL-Sheep Forecasting Model

### 3.1. Overall Framework

To forecast key physiological and behavioral indicators in meat sheep, this study proposes GCL-Sheep (Graph-Enhanced Contextual Long-Range Forecasting for Sheep Farming), a management-context-aware AI model designed for multi-source farm monitoring data. The model is designed to address three practical forecasting challenges in precision sheep farming: associations between barn environment and animal-state indicators, context-dependent interpretation of similar numerical patterns, and possible cumulative or lagged temporal relationships in animal-state sequences.

**Figure 3:**
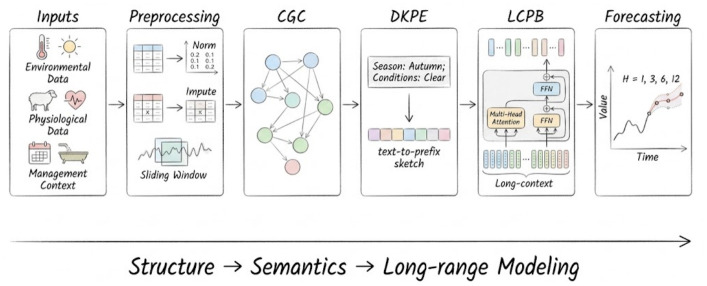
Overall framework of GCL-Sheep.

As shown in Figure 3, GCL-Sheep consists of three core modules: a Cross-Variable Graph Construction (CGC) module, a hierarchical Domain Knowledge Prefix Encoding (DKPE) module, and a Long-Context Prediction Backbone (LCPB). CGC represents structural relationships between environmental variables and physiological/behavioral indicators; hierarchical DKPE introduces farm-, season-, barn-, and event-level management context; and LCPB models the temporal evolution of animal states over extended historical windows. Together, these modules allow the model to generate forecasts that are informed by sensor data, animal-state history, and farm operation information.

The workflow of GCL-Sheep is as follows: First, preprocessed multi-source monitoring sequences are used to construct graph-enhanced representations of environment–animal-state relationships. Second, domain-level background and event-level management context are encoded as hierarchical prefixes and injected into the temporal representation. Third, the long-context backbone learns dependencies across multiple time scales so that delayed associations related to environmental exposure and routine operations can be incorporated into future predictions. Finally, the prediction head maps the learned representations to multi-step forecasts of the target physiological or behavioral indicator [31,32].

Unlike single-level contextual prefixes, the proposed DKPE simultaneously introduces slow-varying background variables and fast-varying operational states. The former are used to anchor the farm–season–barn condition to which the current sample belongs, whereas the latter are used to describe immediate background factors such as feeding, day–night status, manure cleaning, and equipment operation. In this way, the model can use management-context information to reduce confusion between routine operation-related fluctuations and potential stress-related deviations under cross-domain conditions. Functionally, the three modules are tightly connected: CGC organizes variable relationships, DKPE supplements hierarchical semantic constraints, and LCPB performs long-range temporal modeling on structured and contextualized representations, together forming a complete forecasting pipeline [33,34].

At the level of data organization and experimental evaluation, the multi-scenario monitoring data are further organized into farm–season–barn domains, and both in-domain forecasting and cross-domain forecasting settings are adopted in the subsequent experiments to examine the transferability of the proposed framework under unseen scenarios [35,36].

### 3.2. Cross-Variable Graph Construction

To characterize the complex coupling relationships between environmental factors and physiological states during meat sheep farming, we introduce a cross-variable relationship graph into the model. This module represents different monitoring indicators in multivariate time-series forecasting as graph nodes, while edges are used to indicate the association strength between variables. In this way, the structural relationships among variables can be further characterized beyond temporal modeling alone. Let the constructed heterogeneous variable association graph be denoted as G=(V,E), where the node set V represents different types of monitoring variables and the edge set E represents the interaction relationships among variables.

#### 3.2.1. Static Prior Graph Guided by Domain Knowledge

The input features are divided into two groups of nodes: One group is the environmental node set $V_{\mathrm{env}}$, which includes environmental variables such as temperature, relative humidity, ammonia concentration, carbon dioxide concentration, PM_2.5_, PM_10_, and light intensity. The other group is the physiological node set $V_{\mathrm{phys}}$, which includes individual physiological and behavioral indicators such as active duration, rumination duration, feeding duration, intense exercise duration, and body temperature. The total number of nodes in the graph is denoted as

(9) $$N=\;|V_{\mathrm{env}}|\;+\;|V_{\mathrm{phys}}|$$

Previous studies in veterinary science, livestock environmental physiology, and sensor-based sheep behavior monitoring have shown that sheep’s physiological and behavioral states are associated with barn environmental conditions, air-quality exposure, feeding-related behavior, and thermal stress. For example, ammonia exposure has been reported to affect sheep’s feed intake, chewing activity during eating and rumination, respiratory response, and stress-related physiological indicators [37]. Temperature and relative humidity are closely related to heat- or cold-stress conditions and can influence feeding behavior, activity allocation, drinking behavior, and thermoregulatory responses in sheep [6,7]. In addition, particulate matter such as PM_2.5_ and PM_10_ is an important component of livestock-house air quality, and its concentration is affected by ventilation, humidity, and routine farming activities in sheep and goat buildings [38]. Feeding and rumination are also closely related ingestive behaviors and have been widely used as sensor-derived behavioral indicators for sheep health and welfare assessment [1,4,5].

Based on these studies, we introduced domain expert knowledge to construct an initial adjacency matrix $A_{\mathrm{prior}}\in R^{N\times N}$ between the environmental node set $V_{\mathrm{env}}$ and the physiological node set $V_{\mathrm{phys}}$. It should be noted that the static prior graph was constructed as a biologically informed inductive bias rather than as a direct causal verification in the present dataset. The graph topology was mainly constructed according to the following rules. To improve reproducibility, the complete list of prior edges used to initialize $A_{\mathrm{prior}}$, together with their edge type and biological or environmental justification, is provided in Appendix A.

*Cross-Modal Prior Edges:* These edges were used to represent plausible associations between barn environmental exposure and animal-state indicators. Specifically, air-quality variables such as NH_3_ and CO_2_ were connected to feeding- and rumination-related behavioral nodes to represent possible air-quality and ventilation-related influences on ingestive behavior. Temperature and relative humidity were connected to activity- and body-temperature-related nodes to represent the thermal-environment background associated with heat- or cold-stress conditions. PM_2.5_ and PM_10_ were treated as particulate-exposure nodes reflecting barn air-quality conditions.

*Intra-Physiological Prior Edges:* These edges were used to preserve plausible relationships among physiological and behavioral indicators. For example, intense exercise duration was connected with body temperature because high-intensity movement may be accompanied by short-term thermoregulatory changes. Rumination duration and feeding duration were also connected because they are closely related ingestive-behavior indicators and may exhibit coordinated or alternating patterns in time allocation.

*Intra-Environmental Prior Edges:* These edges were used to preserve the covariation structure among environmental variables. For example, temperature and relative humidity often change jointly under barn ventilation and heating conditions, while PM_2.5_ and PM_10_ are both particulate-matter indicators and may show correlated fluctuations under dust exposure and management activities.

During the initialization of $A_{\mathrm{prior}}$, the adjacency weight for each node pair (i,j) satisfying the above prior relationships was set to 1, while the weight for the remaining node pairs without explicitly specified prior relationships was set to 0. Such a sparse prior topology helps narrow the search space for graph structure learning and makes the initial connections among variables more consistent with existing knowledge from sheep environmental physiology, animal behavior, and livestock-house air-quality monitoring. The final graph structure was not determined only by these prior edges; it was further adapted by the data-driven adjacency-learning module during model training. Therefore, the edge list in Appendix A should be understood as the initialization rule for the static prior graph, rather than as a fixed causal graph imposed throughout training.

#### 3.2.2. Dynamic Data-Driven Adjacency Learning

Considering inter-individual differences among meat sheep and micro-level fluctuations in the farming environment, a static graph cannot capture all latent associations. Therefore, we introduce an adaptive learning mechanism based on node embeddings. Specifically, each node i is initialized with a learnable embedding vector $e_{i}\;\in \;R^{d}$. A dynamic association matrix $A_{\mathrm{data}}$ is then generated by computing the similarity among node embeddings:

(10) $$A_{\mathrm{data}}=\mathrm{Softmax}\left(\mathrm{ReLU}(EE^{⊤})\right)$$

where $E\;\in \;R^{N\times d}$ denotes the embedding matrix of all nodes. This module enables the model to automatically mine lagged associations or implicit couplings hidden in the data, such as the influence of specific illumination patterns on the feeding rhythm of meat sheep.

#### 3.2.3. Graph Fusion and Feature Evolution

The final adjacency matrix *A* is obtained by weighted fusion of the prior-knowledge graph and the data-driven graph:

(11) $$A=\alpha A_{\mathrm{prior}}+\left(1-\alpha \right)A_{\mathrm{data}}$$

where α∈[0,1] controls the relative contribution of the static prior graph and the data-driven graph. When α=1, the model relies only on the predefined prior topology; when α=0, the model relies only on the data-driven adjacency matrix. In this study, α was treated as a hyperparameter rather than being fixed arbitrarily. Candidate values {0,0.25,0.5,0.75,1.0} were evaluated using the validation set during preliminary model selection, and α=0.5 was selected because it provided the most stable validation performance and a balanced contribution from prior knowledge and data-driven adjacency learning. After selection, this value was kept fixed in all subsequent test-set evaluation, LODO cross-domain testing, ablation analysis, and few-shot adaptation experiments. The held-out test domains were not used for selecting α. During the graph aggregation stage, we employed a two-layer GCN to perform spatial propagation over the variable-node features at each time step. Let the node feature matrix at time step t be $X_{t}\;\in \;R^{N\times d_{\mathrm{in}}}$, and let the fused adjacency matrix be $A\;\in \;R^{N\times N}$. Then, the graph convolution operation at the l-th layer is defined as follows:

(12) $$H_{t}^{\left(l+1\right)}=\sigma \left({\tilde{D}}^{-1/2}\tilde{A}{\tilde{D}}^{-1/2}H_{t}^{\left(l\right)}W^{\left(l\right)}\right)$$

where $H_{t}^{\left(0\right)}\;=\;X_{t}$, $\tilde{A}\;=\;A\;+\;I$, and $\tilde{D}$ is the corresponding degree matrix. The hidden dimensions of the two GCN layers are both set to 128, and each layer is followed by ReLU activation and dropout (0.1). The final graph-enhanced representation is denoted as $H_{t}^{G}\;\in \;R^{N\times 128}$.

This process allows each variable representation to incorporate related environmental and animal-state information before entering the long-context Transformer, which may improve the stability of forecasting under environmental fluctuations.

### 3.3. Hierarchical Domain Knowledge Prefix Encoding Module

Modeling based solely on numerical sequences may confuse similar temporal patterns that occur under different management backgrounds and scenario conditions. For example, an increase in activity may correspond to routine post-feeding movement or to a potential response under unfavorable environmental conditions. When the model is extended from a single winter barn to multi-farm, multi-season, and multi-barn scenarios, similar numerical fluctuations may have different management or environmental contexts across domains. To alleviate this problem, we designed a hierarchical DKPE module, while jointly introducing domain-level background information and event-level operational information [39].

#### 3.3.1. Hierarchical Context Representation

In this study, the contextual information associated with each input sample is organized into two levels:

(13) $$C=\{C^{\mathrm{dom}},C^{\mathrm{evt}}\}$$

where $C^{\mathrm{dom}}$ denotes the domain-level background information, mainly including slow-varying background factors such as farm, season, and barn identifier or barn type; and $C^{\mathrm{evt}}$ denotes the event-level operational context, mainly including fast-varying background factors such as day–night status, feeding/fasting stage, manure cleaning operations, vaccination, and the on/off status of fans, exhaust equipment, and heating devices. The domain-level context is used to characterize the long-term environmental and operational background in which a sample is situated, whereas the event-level context is used to describe the immediate management conditions within the current input window.

For textual representation, we separately constructed domain-level and event-level descriptions. For example, a domain-level description can be written as follows:

“Domain context: Aksu farm, winter season, enclosed barn, controlled ventilation regime.”

An event-level description can be written as follows:

“Current scenario: nighttime, post-feeding period, fan on, no cleaning, no vaccination.”

It should be noted that these textual descriptions were used as human-readable templates for organizing categorical farm and management context, rather than as free-text inputs to a pretrained language model. In the implementation, each contextual field was first converted into a discrete categorical token. For the domain-level context, farm ID, season, and barn ID or barn type were mapped to trainable embedding vectors. For the event-level context, day–night status, feeding or fasting stage, manure-cleaning state, vaccination state, and equipment operation states were also mapped to trainable embedding vectors. The embeddings belonging to the same context level were concatenated and then projected by a linear layer to generate the corresponding domain-level and event-level prefix representations.

No pretrained language model, such as BERT or a large language model, was used in the DKPE module. Therefore, there was no frozen or fine-tuned external language encoder in this study. The domain-level and event-level embedding tables, together with the projection layers used for prefix generation, were randomly initialized and trained jointly with the forecasting model. This design was adopted to keep the context-encoding process lightweight, reproducible, and directly linked to the structured management records available in farm monitoring data.

Compared with using only single-level event tags, this hierarchical description preserves both the slow-varying scenario background and the fast-varying operational state, allowing the model input to explicitly include both current operational information and the corresponding domain background.

#### 3.3.2. Dual-Level Prefix Generation and Fusion

To inject the above hierarchical context into the time-series forecasting backbone, we separately encoded the domain-level description and the event-level description, and we mapped them into the same hidden space as the forecasting backbone. Let the domain-level prefix encoder and the event-level prefix encoder be denoted by $E_{\mathrm{dom}}(\cdot )$ and $E_{\mathrm{evt}}(\cdot )$, respectively. Then, we have

(14) $$P^{\mathrm{dom}}=E_{\mathrm{dom}}\left(C^{\mathrm{dom}}\right)$$

(15) $$P^{\mathrm{evt}}=E_{\mathrm{evt}}\left(C^{\mathrm{evt}}\right)$$

The two prefix representations are then fused into a unified hierarchical semantic prefix matrix P:

(16) $$P=\mathrm{Fuse}(P^{\mathrm{dom}},P^{\mathrm{evt}})$$

where Fuse(·) denotes the prefix fusion operation, which can be implemented by concatenation followed by linear projection or by weighted combination. Let the total prefix length be k and the hidden dimension be d. Then, the fused prefix representation satisfies

(17) $$P\in R^{k\times d}$$

To ensure that the hierarchical semantic prefix and the graph-enhanced sequential representation lie in the same latent space, we further introduce a linear projection layer to align the scale and dimensionality of P, so that it can participate in the subsequent self-attention computation together with the graph-enhanced representation G in a shared manifold space.

#### 3.3.3. Hierarchical Semantic-Guided Feature Injection Mechanism

The fused hierarchical semantic prefix P is concatenated to the front of the graph-enhanced sequence G, forming the enhanced input representation:

(18) Z=[P;G]

In the subsequent long-context Transformer, the self-attention mechanism treats the prefix part as a semantic reference while modeling temporal dependencies. The domain-level prefix mainly serves as a background anchor, informing the model which farm–season–barn condition the current sample belongs to. The event-level prefix mainly serves as a local contextual controller, informing the model of the immediate operational background within the current time window. In this way, fluctuations in active duration, rumination duration, or body temperature are represented together with the corresponding domain background and operational scenario, rather than being treated only as numerical changes.

This hierarchical semantic injection mechanism plays two roles. On the one hand, in in-domain forecasting, it helps the model use management context to separate routine operation-related fluctuations from potential environment-related deviations in the representation space. On the other hand, in cross-domain transfer, it provides additional background anchors for similar numerical patterns under different farms, seasons, and barns, thereby helping reduce representation inconsistency caused by cross-domain distribution shifts.

### 3.4. Long-Context Prediction Backbone for Cumulative and Lagged Temporal Associations

After graph-structure enhancement and hierarchical semantic conditioning, the resulting augmented feature sequence $H_{\mathrm{aug}}$ is fed into the Long-Context Prediction Backbone. This module is designed to address an important temporal modeling challenge in meat sheep forecasting, namely, how to represent cumulative and lagged associations between environmental fluctuations and animal-state sequences.

#### 3.4.1. Patch-Based Sequence Representation and Compression

To handle extremely long lookback windows without losing local details, the backbone first adopts a patching mechanism. The continuous time series that integrates graph information and prefix semantics is divided into a set of overlapping patches, each of which is treated as an independent token:

(19) $$X_{\mathrm{patch}}=\mathrm{Reshape}\left(H_{\mathrm{aug}}\right)\in R^{P\times (S\cdot d)}$$

where P denotes the number of patches and S denotes the patch stride. This design not only substantially reduces the quadratic computational complexity of the Transformer self-attention mechanism but also enhances the model’s ability to capture subtle fluctuations in physiological indicators by aggregating local temporal information.

#### 3.4.2. Enhanced Time Attention Mechanism

The backbone network in this framework adopts a decoder-only Transformer architecture. Its core component is an enhanced temporal attention mechanism (TimeAttention), which enables unified modeling of the integrated prefix–graph–time token sequence:

(20) $$\mathrm{Output}=\mathrm{Softmax}\left(\frac{(Q_{t}K_{t}^{⊤})⊙M}{\sqrt{d}}\right)V_{t}$$

where M denotes the causal mask, which ensures that the forecasting process strictly follows temporal causality. Through the long-context attention mechanism, the model can capture long-range statistical dependencies—for example, potential associations between earlier environmental fluctuations and later changes in animal-state indicators.

#### 3.4.3. Lagged Physiological Feedback Modeling

Meat sheep’s physiological and behavioral indicators may show delayed variation patterns under changing environmental and management conditions. The LCPB module leverages the global receptive capability of the Transformer and extracts temporal features at different scales through stacked self-attention layers. Lower layers are expected to capture short-term behavioral and body-temperature fluctuations, whereas higher layers are expected to represent lower-frequency temporal patterns over longer historical windows. This hierarchical modeling strategy is intended to help the model separate short-term fluctuations from longer-term temporal patterns under complex farming environments.

#### 3.4.4. Forecasting Head

This study adopts a direct multi-step forecasting strategy. The deep hidden representations output by the backbone network are mapped into the final prediction space through a forecasting head composed of a linear projection layer. To model the future evolution of the target variable, we employ a linear prediction head that maps the deep features generated by the backbone to the sequence of physiological and behavioral indicators of meat sheep over the next H time steps:

(21) $$\hat{Y}=\mathrm{Linear}\left(\mathrm{Flatten}\left(H_{\mathrm{out}}\right)\right)$$

By computing the mean squared error (MSE) between the predicted value $\hat{Y}$ and the ground-truth value Y, together with the proposed distribution alignment loss, the entire framework is jointly optimized in an end-to-end manner.

### 3.5. Loss Function and Multi-Objective Optimization

To enable the GCL-Sheep framework to balance forecasting accuracy and representation-level compatibility between numerical sequences and contextual information, we designed a joint multi-objective loss function. This function consists of two components: a forecasting loss for point-wise prediction error, and a distribution alignment loss for semantic–numerical alignment.

#### 3.5.1. Forecasting Loss

To measure the deviation between the predicted value $\hat{Y}$ and the ground-truth value *Y*, we adopted the mean squared error (MSE) as the primary loss function. Because MSE is relatively sensitive to outliers, it helps the model capture abnormal fluctuations in the target variable under environmental disturbances. Its formulation is as follows:

(22) $$L_{\mathrm{mse}}=\frac{1}{H\cdot C}\sum_{i=1}^{H}\sum_{j=1}^{C}{\left(y_{i,j}-{\hat{y}}_{i,j}\right)}^{2}$$

where *H* denotes the forecasting horizon, and *C* corresponds to the dimensionality of the target variable, with C=1 in this study.

#### 3.5.2. Cross-Modal Distribution Alignment Loss Under Hierarchical Semantic Constraints

Simple feature concatenation may be insufficient to coordinate hierarchical contextual information with time-series numerical representations. To address this issue, we introduce a cross-modal distribution alignment loss to constrain the latent distributions of the graph-enhanced representation and the hierarchical semantic prefix representation. Let $H^{G}$ denote the graph-enhanced sequential representation, and let *P* denote the hierarchical semantic prefix obtained by fusing the domain-level prefix $P^{\mathrm{dom}}$ and the event-level prefix $P^{\mathrm{evt}}$. Their pooled representations over the temporal dimension are computed as follows:

(23) $${\bar{H}}^{G}=\mathrm{Pool}\left(H^{G}\right),\;\bar{P}=\mathrm{Pool}\left(P\right)$$

We then construct the distribution alignment loss using the Kullback–Leibler (KL) divergence:

(24) $$L_{\mathrm{align}}=D_{\mathrm{KL}}\left(\mathrm{softmax}\left(\frac{{\bar{H}}^{G}}{\tau }\right)\;∥\;\mathrm{softmax}\left(\frac{\bar{P}}{\tau }\right)\right)$$

where τ is the temperature parameter. In this study, τ was set to 1.0. This value was selected according to preliminary validation and training stability, because it provided a stable alignment signal without excessively smoothing or sharpening the latent distributions. This loss term encourages the model to learn time-series latent representations that are compatible with the hierarchical context under a given domain background and operational condition, thereby serving as an auxiliary regularization term under non-stationary and cross-domain forecasting settings.

#### 3.5.3. Joint Optimization Objective

Finally, the entire framework is trained through end-to-end backpropagation, and the overall loss function $L_{\mathrm{total}}$ is defined as follows:

(25) $$L_{\mathrm{total}}=\lambda_{1}L_{\mathrm{mse}}+\lambda_{2}L_{\mathrm{align}}.$$

where $\lambda_{1}$ and $\lambda_{2}$ are balancing coefficients for the forecasting loss and the alignment loss, respectively. In this study, we set $\lambda_{1}=1.0$ and $\lambda_{2}=0.1$. The forecasting loss was kept as the dominant optimization objective, while the alignment loss was used as an auxiliary regularization term. The values of $\lambda_{2}$ and τ were selected through preliminary validation from candidate settings and were then fixed for all subsequent model comparison, cross-domain testing, ablation analysis, sensitivity analysis, and few-shot adaptation experiments. No test-set or held-out target-domain samples were used to select these hyperparameters.

This multi-objective optimization strategy enables GCL-Sheep to optimize forecasting accuracy while using hierarchical context information as an auxiliary representation-level constraint. Therefore, the alignment term should be interpreted as a computational regularization strategy for context-aware forecasting, rather than as evidence that the model verifies biological mechanisms or causal physiological relationships.

## 4. Experimental Design and Results

### 4.1. Baseline Models for Comparison

To ensure a fair comparison, all baseline models were evaluated under the same data-splitting protocol, forecasting horizons, target-specific forecasting setting, and input feature scope as GCL-Sheep. Specifically, all models used the same preprocessed hourly input features, including barn environmental variables, historical physiological/behavioral indicators, and management-context variables. Unless otherwise specified, the default lookback window was set to L=96 h for all sequence-based models, and the forecasting horizons were set to H=1,3,6, and 12 h. For XGBoost, the historical window was flattened into a fixed-length feature vector. For neural-network baselines, the original temporal sequence structure was preserved. All baseline hyperparameters were selected using the validation set only, and the test set was not used for model selection.

- **XGBoost**: An efficient algorithm based on gradient boosting decision trees (GBDT). By integrating multiple weak regressors with regularization techniques, it has demonstrated strong robustness in time-series forecasting tasks that rely heavily on feature engineering.
- **LSTM**: A classic variant of recurrent neural networks (RNNs). By introducing gating mechanisms, it effectively alleviates the vanishing-gradient problem in long-sequence training and is well suited for capturing long-term dependencies in time series.
- **TCN**: Adopts causal convolution and dilated convolution architectures, achieving a large receptive field while preserving temporal causality. Compared with RNN-based models, it also offers better parallel computational efficiency.
- **Transformer**: Completely discards recurrent structures and relies entirely on the self-attention mechanism to capture global dependencies within sequences. It serves as a fundamental backbone for current long-range time-series forecasting models.
- **PatchTST**: Introduces a patching strategy to enhance local temporal representation and, together with a channel-independent design, improves the efficiency of Transformer-based long-sequence forecasting.
- **Time-LLM**: A cross-modal time-series model that reprograms time-series data into prompt-like representations, thereby leveraging pretrained language-model representations for forecasting.

The main implementation settings of the baseline models are summarized in Table 6. The same chronological training, validation, and test splits were used for GCL-Sheep and all baselines. For in-domain evaluation, samples within each domain were split at a 7:1:2 chronological ratio. For LODO evaluation, the same source-domain training and held-out target-domain testing protocol was applied to all models.

### 4.2. Evaluation Metrics

To evaluate the forecasting performance of GCL-Sheep for meat sheep’s physiological and behavioral indicators, this study adopts the mean absolute error (MAE), root-mean-square error (RMSE), and the coefficient of determination (R^2^) as evaluation metrics. MAE describes the average absolute deviation between predicted and observed values, RMSE gives stronger penalties to larger prediction errors, and R^2^ measures the overall goodness of fit relative to the variation in the target variable. Because prediction targets have different physical units and numerical ranges, continuous targets were standardized using training-set statistics. Unless otherwise stated, MAE and RMSE values for model and setting comparisons are reported on the standardized target scale. Inverse-standardized MAE and RMSE values in original units are additionally reported where appropriate in the corresponding results tables.

The three metrics are computed as follows:

(26) $$\mathrm{MAE}=\frac{1}{n}\sum_{i=1}^{n}\left|y_{i}-{\hat{y}}_{i}\right|$$

(27) $$\mathrm{RMSE}=\sqrt{\frac{1}{n}\sum_{i=1}^{n}{\left(y_{i}-{\hat{y}}_{i}\right)}^{2}}$$

(28) $$R^{2}=1-\frac{\sum_{i=1}^{n}{\left(y_{i}-{\hat{y}}_{i}\right)}^{2}}{\sum_{i=1}^{n}{\left(y_{i}-\bar{y}\right)}^{2}}$$

where $y_{i}$ denotes the ground-truth observation, ${\hat{y}}_{i}$ denotes the model prediction, $\bar{y}$ denotes the mean of the ground-truth values, and n denotes the total number of test samples. Through the joint analysis of these three metrics, overall model performance is assessed in terms of error magnitude, sensitivity to large deviations, and goodness of fit under the same experimental protocol.

### 4.3. Experimental Settings

#### 4.3.1. In-Domain Forecasting Setting

The in-domain forecasting experiments were designed to evaluate the multi-step forecasting capability of the model for key physiological and behavioral indicators of meat sheep under the same farm–season–barn condition. Following the supervised sample construction procedure defined in Section 2, the samples within each domain were split into training, validation, and test sets in chronological order, at a ratio of 7:1:2, so as to avoid temporal leakage caused by random partitioning. Sliding-window samples were generated independently within each subset and did not cross the boundaries between the training, validation, and test sets. For a given target variable, the model was trained only on the historical window containing environmental features, historical individual-state features, and management context features, and it was used to predict the target values over multiple future time steps.

Unless otherwise specified, the default lookback window length was set to L=96, and the forecasting horizon was selected from H∈{1,3,6,12}. This setting is consistent with the problem formulation described above and with the subsequent multi-step forecasting experiments. Here, L=96 is intended to cover cross-day historical information and possible delayed temporal associations in the monitoring data, whereas different values of *H* correspond to short-term, medium-term, and relatively long-term forecasting tasks. All deep-learning models were compared under the same hardware environment and training strategy to ensure the comparability of the experimental results.

During training, we used the Adam optimizer with an initial learning rate of $1\times 10^{-4}$ and a batch size of 32, together with a cosine decay learning-rate schedule for parameter updating. To prevent overfitting, early stopping was adopted with a patience of 10. The hidden dimension of the model was uniformly set to 128, and the patch length was set to 16. All deep models were trained on an NVIDIA RTX 4090 GPU.

The validation set was used only for model selection, early stopping, and hyperparameter tuning, whereas the test set was used only for final performance evaluation. No test samples or held-out target-domain samples were used during model selection. To assess the stability of the results, each experiment was independently repeated three times using random seeds 41, 42, and 43. Unless otherwise specified, all quantitative performance results in Section 4.4, Section 4.5 and Section 4.6 are reported as the mean ± standard deviation across the three independent runs. For figures with error bars, the plotted points represent the mean values and the error bars denote the standard deviation across the three runs. This reporting strategy was applied consistently to model comparison, cross-domain evaluation, few-shot adaptation, lookback-window analysis, ablation studies, and hyperparameter sensitivity analyses.

The approximate number of trainable parameters for each model is summarized in Table 7. For Time-LLM, only the trainable reprogramming and forecasting layers were counted, because the pretrained language-model backbone was kept frozen. XGBoost is a tree-based model and, therefore, does not have trainable neural-network parameters in the same sense as the deep-learning baselines.

#### 4.3.2. Cross-Domain Forecasting Setting and Leave-One-Domain-Out Protocol

To evaluate the generalization ability of the model under unseen scenarios, we further conducted cross-domain forecasting experiments using the Leave-One-Domain-Out (LODO) protocol. According to the definition in Section 2, each domain is jointly determined by farm, season, and barn, denoted as d=(f,s,b). Let the integrated domain set be denoted as $D=\{d_{1},d_{2},\ldots ,d_{M}\}$. Then, in the m-th round of cross-domain experiments, one domain $d_{m}$ is selected as the target-domain test set, while the remaining M-1 domains are merged to form the source-domain training set:

(29) $$D_{m}^{src}=D\setminus \left\{d_{m}\right\}$$

The model was trained and validated only on the source-domain data, and it was directly tested on the target domain after training, without any target-specific retraining or parameter updating. This setting was intended to examine whether the learned variable-structure representations and contextual semantic representations can be transferred to unseen farm–season–barn combinations, rather than merely fitting local temporal patterns within a single scenario.

Under the LODO protocol, the source-domain data were still split into training and validation sets in chronological order for model selection, hyperparameter tuning, and early stopping. The target-domain samples remained completely independent in that experimental round and were used only for final testing. In addition to zero-shot cross-domain testing, we further introduced a few-shot adaptation setting. After source-domain training was completed, a small number of temporally consecutive sample windows were drawn from the target domain as a lightweight adaptation set, while the remaining target-domain samples continued to serve as the test set. This setting was designed to simulate the practical deployment scenario in which only a limited amount of local observational data is available during the early stage of a new barn’s operation. Through this design, we can further analyze the performance transition of the model from zero-shot cross-domain generalization to rapid adaptation with limited target-domain samples.

To ensure consistency between the in-domain and cross-domain settings, the cross-domain experiments also adopted the default lookback window length of L = 96 h and the multi-step forecasting horizons H∈{1,3,6,12}, together with the same training hyperparameter configuration as used in the in-domain experiments. The subsequent experiments report the performance of the model under in-domain forecasting, zero-shot cross-domain forecasting, and few-shot adaptation, respectively, and further compare how the forecasting error changes as the prediction horizon increases, so as to analyze the stability of the model under domain-shift conditions [40].

#### 4.3.3. Implementation Details and Hyperparameter Rationale

To improve the reproducibility of the proposed model, the key implementation settings and hyperparameter-selection rationale are summarized in Table 8. The hyperparameters were determined according to three criteria: consistency with commonly used settings in graph-based and Transformer-based time-series models, validation-set performance in preliminary experiments, and computational stability under the available sample size. After selection, all hyperparameters were kept fixed across the model comparison, ablation, cross-domain, and few-shot adaptation experiments.

The graph fusion weight α was selected from {0,0.25,0.5,0.75,1.0}, and the value of 0.5 was adopted because it provided the best validation performance and achieved a balance between the prior-knowledge graph and the data-driven adjacency matrix. The numbers of GCN layers and hidden dimensions were set to two layers and 128 dimensions, in order to provide sufficient cross-variable representation capacity while avoiding excessive model complexity and over-smoothing. A dropout rate of 0.1 was used as a mild regularization strategy to reduce overfitting. For the long-context backbone, a patch length of 16 h and a patch stride of 8 h were used to reduce the effective sequence length while preserving local temporal continuity. For the distribution alignment term, KL divergence was used to constrain the latent distribution of graph-enhanced numerical representations and hierarchical semantic prefixes. The loss weights were set to $\lambda_{1}=1.0$ and $\lambda_{2}=0.1$, and the temperature parameter was set to τ=1.0 after preliminary validation, so that the forecasting loss remained dominant while the alignment loss acted as an auxiliary regularization term.

### 4.4. Animal-State Forecasting Results and Analysis

#### 4.4.1. Comparison of In-Domain Multi-Step Forecasting Performance

To evaluate the adaptability of the model to different forecasting horizons under the in-domain setting, this experiment fixed the historical lookback window at L = 96, split the samples within each domain into training, validation, and test sets in chronological order, and compared the performance of GCL-Sheep with multiple baseline models under different forecasting horizons H. The experimental results are presented in Table 9. As shown in Table 9, under the in-domain forecasting setting, the MAE and RMSE of all models generally increase as the forecasting horizon becomes longer, whereas R^2^ gradually decreases. This indicates that error accumulation and weakened fitting ability are common phenomena in multi-step forecasting tasks. It also suggests that, as the prediction horizon extends, the model can no longer rely solely on the local inertia of short-term sequences but must more effectively characterize the complex relationships among environmental variables, behavioral states, and temporal context. Under this condition, GCL-Sheep achieves the best results across all forecasting horizons, indicating that its advantage is not limited to short-term forecasting but can also be maintained under longer prediction horizons.

A further comparison across different horizons shows that, under short-horizon conditions, the performance differences among models are relatively small, suggesting that forecasting at this stage mainly depends on local variation information from the most recent time steps. However, as the forecasting horizon continues to increase, the performance gap among models becomes progressively more pronounced. In particular, under long-horizon conditions, GCL-Sheep still maintains lower errors and higher R^2^ values. This result indicates that the proposed method has a stronger ability to control error propagation in long-range forecasting, rather than merely improving short-term point-to-point fitting. In other words, when the task shifts from extrapolating recent trends to predicting behavioral states further into the future, the advantages of GCL-Sheep in modeling long-range dependencies and cross-variable relationships become more evident. This result also provides an in-domain reference for the subsequent cross-domain forecasting analysis.

**Figure 4:**
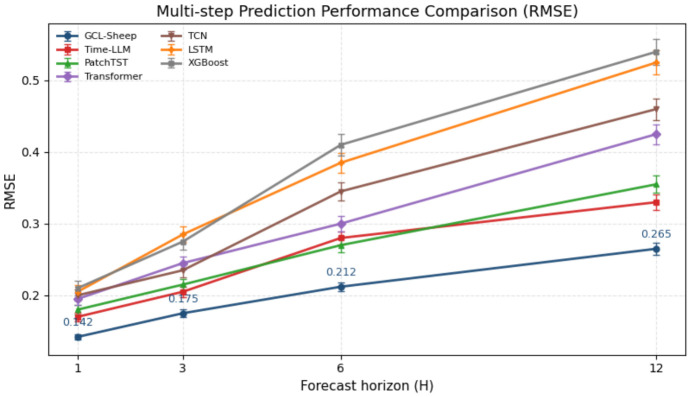
Comparison of RMSE curves of different models under varying forecasting horizons. For each model and forecasting horizon, the plotted value represents the mean RMSE averaged across the five farm–season–barn domains and three independent runs with random seeds 41, 42, and 43. Error bars denote the standard deviation across the three independent runs.

As shown in Figure 4, the RMSE of all models continuously increases as the forecasting horizon becomes longer; however, the curve of GCL-Sheep remains consistently at the lowest position overall, with a relatively gentler growth slope. This indicates that, as the prediction span expands, the performance of GCL-Sheep degrades more slowly, suggesting better long-horizon forecasting stability. This trend is consistent with the design motivation of GCL-Sheep, in which cross-variable graph representation, hierarchical management-context input, and long-context temporal modeling are jointly used to support multi-step forecasting. Because this analysis is based on predictive performance rather than direct physiological intervention tests, the results should be interpreted as forecasting-level evidence that the proposed representation strategy improves long-horizon stability.

Taken together, the results in Figure 4 and Table 9 show that GCL-Sheep consistently achieves lower error levels and more stable performance trends across different forecasting horizons. This indicates that the proposed method is more suitable for multi-step forecasting of active duration in meat sheep, and that its advantages are particularly evident in medium- and long-horizon forecasting and management-decision support settings. Because this study evaluates predictive accuracy rather than clinical or welfare-defined event detection, these results should be interpreted as retrospective forecasting evidence rather than as validation of a welfare-threshold-based early-warning system.

#### 4.4.2. Comparison of Cross-Domain Generalization Performance

To evaluate the generalization ability of the model under unseen scenarios, we compared the cross-domain forecasting performance of GCL-Sheep with that of all baseline models under the Leave-One-Domain-Out (LODO) protocol. In each experiment, one farm–season–barn domain was selected as the target test set, while the remaining domains were used as the source-domain training set. Table 10 presents the average cross-domain forecasting results for active duration under the setting of H=12. In addition to the averaged LODO comparison, we further report the performance of GCL-Sheep for each left-out domain to examine whether the model generalizes consistently across different farm, season, and barn conditions.

In the cross-domain forecasting analysis, GCL-Sheep achieved the best overall performance among the compared models. The model obtained a cross-domain R^2^ of 0.792, showing a moderate decrease compared with its in-domain performance of 0.821. This result suggests that the combination of variable-structure representation and hierarchical context encoding helps the model maintain predictive performance under farm–season–barn domain shifts. Rather than demonstrating biological causality, this finding provides computational evidence that incorporating environmental, animal-state, and management-context information can improve cross-domain forecasting stability.

Compared with the second-best model, Time-LLM, GCL-Sheep improved R^2^ by 0.077 under the cross-domain setting. When domain shift occurred in the farming environment, baseline models such as PatchTST and Transformer showed larger performance degradations, whereas GCL-Sheep maintained relatively higher forecasting accuracy. These results indicate that the proposed model has potential for cross-scenario deployment in precision sheep farming, while further on-farm validation is still needed before drawing stronger biological or management conclusions.

To determine whether the averaged LODO performance was dominated by a specific target domain, Table 11 reports the per-domain LODO results of GCL-Sheep. Each row corresponds to one LODO round, in which the listed domain was held out as the target-domain test set and the remaining four domains were used as source domains for training and validation. The results show that GCL-Sheep maintained relatively stable performance across the five farm–season–barn target domains, although the Aksu autumn domain showed slightly lower accuracy, indicating that geographic and seasonal differences still affected cross-domain transferability.

The per-domain results indicate that the average LODO performance did not depend on a single favorable target domain. The model performed relatively well in both the Daqing and Aksu domains, but the slightly lower performance in D4 suggests that cross-domain forecasting remains more challenging under larger geographic and seasonal differences. Therefore, the averaged LODO result should be interpreted together with the per-domain results when evaluating cross-scenario generalization.

#### 4.4.3. Few-Shot Adaptation Analysis

To further examine the model’s ability to rapidly adapt under cold-start conditions in a new scenario, this study introduces a small number of temporally consecutive samples from the target domain for lightweight adaptation on top of the zero-shot cross-domain forecasting setting, and we analyze the effects of different sample proportions on forecasting performance. Table 12 presents the results of the few-shot adaptation experiments for active duration under the forecasting horizon of H = 12.

The results show that adding a limited proportion of target-domain samples was associated with gradual improvements in forecasting performance. Specifically, the R^2^ increased from the cross-domain baseline of 0.792 to 0.830, while both MAE and RMSE decreased continuously. This suggests that a small amount of local data can help the model adjust to farm-location, seasonal-climate, and management-practice differences, although this should be interpreted as forecasting-level adaptation rather than full correction of the underlying domain shift. Notably, when only 10% of the target-domain samples were introduced, the model achieved an R^2^ of 0.818, which was close to the average level of in-domain forecasting. This result suggests that GCL-Sheep has potential for lightweight target-domain adaptation during the early deployment stage of new barns, where large-scale historical data are often unavailable.

#### 4.4.4. Impact of Lookback Window Length

To examine whether longer historical context improves forecasting performance under the in-domain setting, this experiment fixed the forecasting horizon at H = 6 and varied the historical lookback window length L. As shown in Table 13, under the in-domain forecasting setting, the model performance improves continuously as the lookback window increases from 24 h to 96 h: MAE decreases from 0.195 to 0.154, RMSE decreases from 0.268 to 0.212, and R^2^ increases from 0.805 to 0.884. This indicates that appropriately increasing the amount of historical information helps the model better capture the variation trend of the target variable. For active duration in meat sheep, the forecasting target may be influenced by previous environmental fluctuations, management events, and delayed statistical associations in animal-state sequences. Shorter historical windows mainly reflect local inertia and short-term fluctuations, whereas windows of 48–96 h can include more complete information on day–night rhythms, cross-day variation patterns, and delayed associations between environmental and animal-state variables. This trend also suggests that longer historical context can provide useful information for forecasting under subsequent cross-domain conditions.

**Figure 5:**
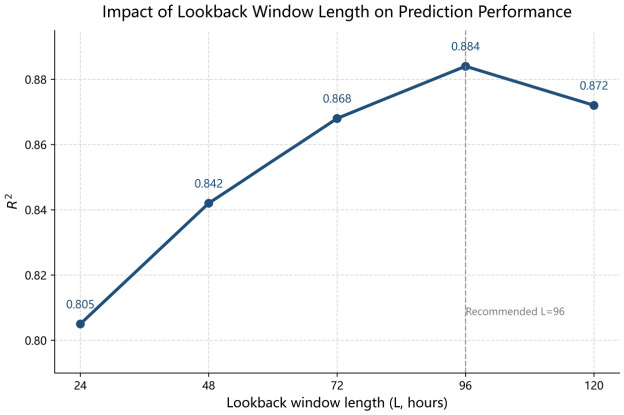
Variation curve of $R^{2}$ for GCL-Sheep under different historical lookback window lengths (H=6).

Combined with the results shown in Figure 5, the model’s R^2^ generally exhibits a trend of first increasing and then decreasing as the historical window length increases, reaching its peak at L = 96. This indicates that an appropriate historical context length exists for the current forecasting task. When the input window covers approximately 2–4 days of temporal context, the model can use more cross-day information than short-window settings. However, as the window continues to expand, the effective gains brought by additional historical information begin to diminish. In other words, the benefit of long context depends on whether the added history contains task-relevant information rather than simply increasing the input length.

It should also be noted that when the historical window is further increased from 96 h to 120 h, the model performance shows a slight decline, with MAE and RMSE rising to 0.161 and 0.218, respectively, and R^2^ decreasing to 0.872. This suggests that longer historical information is not always better, and that an excessively long input window may introduce more weakly related or redundant information, thereby affecting the model’s ability to identify the dominant variation trends. Although the patching mechanism in the LCPB allows the model to remain generally stable under longer input conditions, without showing obvious training instability, the forecasting results indicate that 120 h exceeds the relatively suitable historical information range for the current task.

Overall, L = 96 achieved a favorable balance among error control, trend fitting, and computational cost, and it was therefore adopted as the standard historical window in the subsequent experiments. This result supports the empirical value of using cross-day historical information for medium-term forecasting in this dataset, but it should not be interpreted as direct proof of a specific physiological lag mechanism.

#### 4.4.5. Comparison Between Ground Truth and Predictions in Typical Scenarios

**Figure 6:**
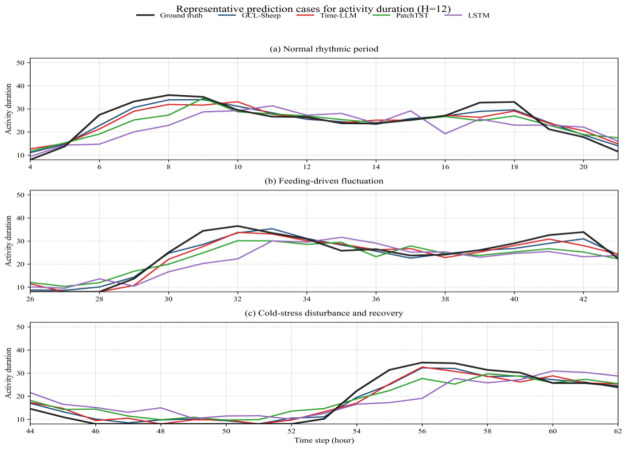
Comparison between ground-truth and predicted activity duration of meat sheep under typical scenarios: (**a**) normal rhythmic period; (**b**) feeding-driven fluctuation period; (**c**) low-temperature environmental-disturbance and recovery period. The period in panel (**c**) was defined using barn temperature records and management logs, rather than clinical cold-stress diagnosis.

To further compare the forecasting performance of different models during temporal evolution, we selected active duration as a representative target variable and, under the in-domain forecasting setting with a forecasting horizon of H=12, chose three typical scenarios from the test set, namely, the normal rhythmic period, the feeding-driven fluctuation period, and the low-temperature environmental-disturbance and recovery period. The corresponding comparison curves between ground-truth values and predicted values are shown in Figure 6. Compared with the overall statistical results based on MAE, RMSE, and $R^{2}$ presented above, these curves provide a more intuitive view of the models’ tracking ability at local peaks, troughs, turning points, and recovery phases.

The low-temperature environmental-disturbance and recovery period was defined using objective environmental and management records rather than clinical diagnosis of cold stress. Specifically, this period was selected when the barn temperature decreased below 5 °C for at least three consecutive hours and the management log recorded heater activation or ventilation adjustment, followed by a recovery stage in which the barn temperature gradually increased above 8 °C within the subsequent observation window. Therefore, this scenario should be interpreted as an operationally defined environmental-disturbance case for evaluating local forecasting behavior, rather than as a validated welfare or clinical cold-stress event.

As shown in Figure 6a, during the normal rhythmic period, all models are able to track the overall variation trend of active duration relatively well, although differences still exist in local peaks and descending phases. Among them, GCL-Sheep is the closest to the ground truth, showing better synchronization during the rise in activity level, the occurrence of peaks, and the subsequent decline. Time-LLM captures the overall direction of change reasonably well, but it still exhibits slight deviations at certain peak positions. PatchTST responds more conservatively to local peaks, showing a certain smoothing effect, whereas LSTM exhibits more obvious lag.

As shown in Figure 6b, during the feeding-driven fluctuation period, active duration rises and falls markedly within a short period, which places higher demands on the model’s ability to characterize rapid local changes. In this scenario, GCL-Sheep still follows the overall contour well and remains relatively consistent with the ground truth during both the peak formation and decline processes. In contrast, Time-LLM still shows some deviation in peak magnitude and descending slope, PatchTST exhibits more pronounced compression of local peaks, and LSTM is more susceptible to smoothing and lag effects.

Figure 6c further illustrates the performance differences among models during the low-temperature environmental-disturbance and recovery period. In this scenario, active duration first declines sharply and then gradually recovers, resulting in a more complex temporal pattern. It can be seen that GCL-Sheep tracks both the abrupt drop and the recovery process more closely to the ground truth, showing better adaptability at the recovery onset, the rising slope, and the subsequent stabilization phase. Time-LLM captures the overall direction of change, but it still exhibits some deviation during the recovery stage. PatchTST responds relatively slowly to abrupt changes, while LSTM shows the most pronounced lag during both the decline and recovery phases.

Overall, the comparison between ground-truth values and predicted values in typical scenarios from the in-domain test set further supports the forecasting performance of GCL-Sheep. The model is able to track changes in active duration under normal rhythmic conditions and also maintains relatively stable prediction performance in more complex scenarios such as feeding-driven fluctuations and environmental-disturbance recovery. This observation is broadly consistent with the quantitative results reported above for in-domain multi-step forecasting performance.

#### 4.4.6. Cross-Variable Forecasting Consistency Analysis

**Figure 7:**
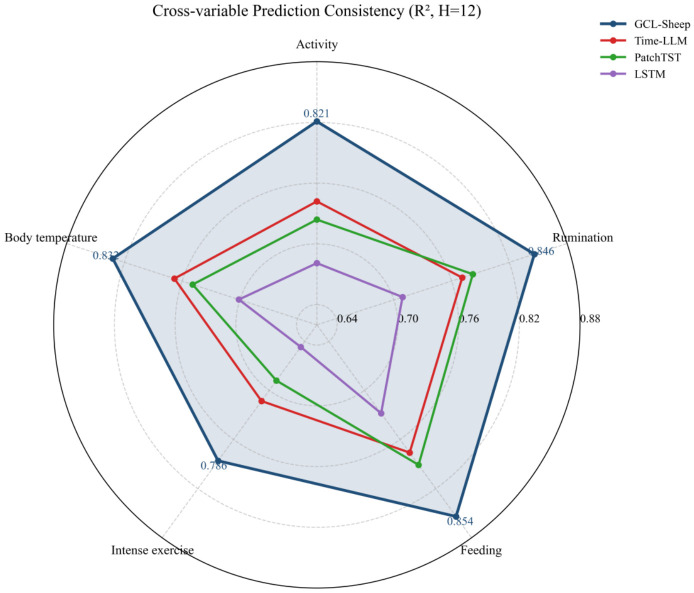
Radar chart of predictive performance ($R^{2}$) across different target variables.

To examine the adaptability of the model across different target variables, we selected five representative indicators—namely, active duration, rumination duration, feeding duration, intense exercise duration, and body temperature—under the in-domain forecasting setting with a forecasting horizon of H=12, and we plotted a radar chart of cross-variable forecasting performance using $R^{2}$ as the evaluation metric, as shown in Figure 7. The comparison models include Time-LLM, PatchTST, and LSTM.

To provide a more complete quantitative evaluation, MAE and RMSE were also calculated for all five target indicators after inverse standardization. The four duration-related indicators are reported in min h^−1^, and body temperature is reported in °C. The corresponding MAE, RMSE, and $R^{2}$ values of GCL-Sheep are summarized in Table 14.

From the overall contour of the radar chart, it can be observed that GCL-Sheep achieves the highest $R^{2}$ on all five prediction targets, demonstrating good cross-variable stability. Specifically, the $R^{2}$ values of GCL-Sheep for predicting active duration, rumination duration, feeding duration, intense exercise duration, and body temperature are 0.821, 0.846, 0.854, 0.786, and 0.832, respectively, and the enclosed area of its radar chart is also clearly larger than those of the other models. This indicates that the proposed framework not only performs well on a single target but also maintains relatively balanced forecasting performance across multiple behavioral and physiological indicators.

As shown in Table 14, GCL-Sheep achieved relatively low MAE and RMSE across all five indicators. Feeding duration and rumination duration showed relatively higher $R^{2}$ values and lower prediction errors, whereas intense exercise duration had the lowest $R^{2}$, indicating that sparse and irregular behavioral events remained more difficult to forecast. Reporting the error metrics in their original units also helps clarify the practical magnitude of the prediction errors.

From the perspective of model architecture, the joint effect of cross-variable relationship modeling, hierarchical contextual encoding, and long-context learning helps enhance the overall representation ability of the model for multi-source temporal information, thereby improving its adaptability across different forecasting tasks.

From the perspective of forecasting difficulty across indicators, feeding duration and rumination duration are generally easier to fit with higher accuracy, whereas intense exercise duration consistently yields the lowest forecasting performance. This suggests that different variables differ substantially in temporal regularity, event sparsity, and noise sensitivity. Feeding and rumination behaviors usually exhibit stronger intra-day rhythmicity and continuity, making them easier for the model to capture. In contrast, intense exercise duration is more sparse and irregular in the recorded sequence, which places higher demands on the model’s ability to represent short-term fluctuations. Even so, GCL-Sheep still achieves an $R^{2}$ of 0.786 on this indicator, outperforming Time-LLM, PatchTST, and LSTM, which indicates that the proposed method is relatively more effective in modeling complex and non-stationary behavioral variables.

A further comparison among the baseline models shows that Time-LLM and PatchTST each exhibit different strengths across variables. The former performs relatively better in predicting active duration, intense exercise duration, and body temperature, whereas the latter performs better on rumination duration and feeding duration, indicating that different models do not adapt equally well to different variable types. However, the radar-chart contours of both methods still show a certain degree of imbalance, suggesting that their cross-variable forecasting stability still has room for improvement. In contrast, LSTM yields relatively lower $R^{2}$ values across all five indicators, especially showing a more pronounced decline in predicting intense exercise duration, which also reflects the limitations of traditional recurrent networks in complex multivariate scenarios and medium- to long-range dependency modeling. Overall, the results in Figure 7 further support the performance of GCL-Sheep in cross-variable forecasting tasks. The model not only outperforms several representative baselines in overall accuracy but also exhibits better balance and stability across different target variables. This indicates that the proposed method can model associations among environmental signals, individual states, and behavioral patterns in a more stable manner for multi-indicator forecasting. This result also provides a basis for subsequent multi-indicator generalization analysis under cross-domain conditions.

### 4.5. Ablation Analysis of Model Components

To further investigate the specific contribution of each core component of GCL-Sheep to the overall performance under the in-domain forecasting setting, we designed and conducted an ablation study, which we report in this section. We took forecasting horizons of H=6 and H=12 as the evaluation settings, fixed the historical lookback window at L=96, and constructed three model variants for comparative analysis. The specific design logic of each variant and the corresponding performance comparison results are presented in Table 15.

As shown in Table 15, the full GCL-Sheep model consistently achieved the lowest MAE and RMSE and the highest $R^{2}$ at both H=6 and H=12. When the CGC module was removed, the H=12 RMSE increased from 0.265±0.007 to 0.315±0.013, and $R^{2}$ decreased from 0.821±0.008 to 0.765±0.018, indicating that graph-based variable representation contributed to forecasting performance. Removing hierarchical DKPE led to the largest decline in $R^{2}$ at H=12, suggesting that management-context information was useful for long-horizon prediction under the current experimental setting. Removing the patching mechanism also increased the forecasting error, indicating that local temporal compression in LCPB helped preserve useful information from the 96 h historical window. These results provide empirical, prediction-level support for the contribution of each module, but they should not be interpreted as independent evidence of biological mechanisms, causal relationships, or semantic superiority. In addition to the module-level ablation experiments, we further conducted an input-source ablation analysis to examine the contribution of different types of monitoring information. Three additional variants were constructed: (1) w/o environmental variables, in which temperature, relative humidity, NH_3_, CO_2_, PM_2.5_, PM_10_, and light-intensity features were removed from the input; (2) w/o management-context variables, in which day–night status, feeding or fasting stage, manure cleaning, vaccination, and equipment-operation states were removed; and (3) w/o animal-history variables, in which historical active duration, rumination duration, feeding duration, intense exercise duration, and body temperature were removed from the input window, while the target sequence remained unchanged. The same training, validation, and test splits were used as in the full model (Table 16).

The input-source ablation results show that removing any information source decreased the forecasting performance, indicating that environmental exposure, management context, and animal-history information all contributed to the prediction task. Among the three input groups, removing animal-history variables caused the largest degradation, suggesting that previous animal-state trajectories provide the most direct information for short- and medium-term forecasting. Removing environmental variables also reduced performance, especially at H=12, indicating that barn exposure information provided useful background for longer-horizon prediction. Removing management-context variables led to a moderate performance decline, which suggests that routine operation records helped the model interpret similar numerical patterns under different farm-management conditions. These results are interpreted from the perspective of predictive performance and should not be taken as independent evidence of causal biological relationships.

### 4.6. Hyperparameter Sensitivity Analysis

#### 4.6.1. Sensitivity to the Graph Fusion Weight *α*

To analyze the fusion strategy between the prior-knowledge graph and the data-driven graph in the Cross-Variable Graph Construction (CGC) module, we further conducted a sensitivity experiment on the graph fusion weight α. Unless otherwise specified, all of the following hyperparameter sensitivity experiments were performed under the in-domain forecasting setting. As defined in the corresponding equation, the fused adjacency matrix was obtained by weighting the prior graph $A_{\mathrm{prior}}$ and the data-driven graph $A_{\mathrm{data}}$, where α controls the contribution of prior knowledge to the graph structure. When α=0, the model relies entirely on data-driven relationships; when α=1, the model uses only the prior-knowledge graph. To compare the effects of different fusion strategies on forecasting performance, we set α∈{0,0.25,0.5,0.75,1.0} in the active duration forecasting task, while fixing the historical lookback window at L = 96 and the forecasting horizon at H = 12. All other parameters were kept the same as those in the main experiments.

As shown by the results in Table 17, as α increases from 0 to 0.5, the model’s MAE and RMSE gradually decrease, while R^2^ correspondingly improves. However, when α is further increased to 0.75 and 1.0, the model performance declines. The best results are achieved at α=0.5, where the MAE, RMSE, and R^2^ are 0.188, 0.265, and 0.821, respectively. Compared with the purely data-driven setting at α=0, this configuration yields lower forecasting error and better fitting performance; compared with the pure prior-graph setting at α=1, α=0.5 also shows better overall results. This indicates that relying on only a single source of graph structure is insufficient to achieve optimal performance.

When α=0, the model constructs the variable graph based entirely on statistical relationships in the data. Although this strategy can reflect certain dynamic coupling relationships, it is also more vulnerable to noise fluctuations, short-term spurious correlations, and non-stationary disturbances. In contrast, when α=1, the model depends entirely on the manually specified prior topology. Although such a graph structure is more directly informed by prior domain knowledge, it is less adaptive to the data-dependent associations induced by individual differences, environmental fluctuations, and stage-dependent changes in real farming processes. The experimental results show that the intermediate fusion range generally outperforms the two extreme settings, with α=0.5 achieving the best performance. This suggests that, for the current task, there exists an appropriate balance between prior-knowledge constraint and data-adaptive modeling. In other words, the prior graph provides predefined domain-informed associations among environmental variables and animal-state indicators, whereas the data-driven graph supplements sample-dependent association information. Their combination may therefore be more suitable for forecasting under complex multivariate farm-monitoring conditions.

#### 4.6.2. Sensitivity to Prefix Length *k*

To examine the influence of prefix length on model performance in the hierarchical Domain Knowledge Prefix Encoding (DKPE) module, we conducted a sensitivity experiment on the total prefix length k. According to the method design, the domain-level background information and the event-level operational context were first encoded separately and then fused into a unified hierarchical semantic prefix representation P, which was subsequently concatenated with the graph-enhanced time-series feature sequence before being fed into the long-context forecasting backbone. The parameter k controls the total length of the fused semantic prefix. To compare how the model utilizes management-context information under different prefix lengths, we set k∈{0,4,8,12,16} in the active duration forecasting task, while fixing the historical lookback window at L = 96 and the forecasting horizon at H = 12. All other parameters were kept the same as those in the main experiments.

As shown in Table 18, as the prefix length increases from 0 to 8, the model’s MAE and RMSE gradually decrease, while R^2^ correspondingly improves. However, when k is further increased to 12 and 16, the model performance shows a slight decline. The best results are achieved at k = 8, where the MAE, RMSE, and R^2^ are 0.188, 0.265, and 0.821, respectively. This result indicates that an appropriate amount of hierarchical contextual prefix information is associated with better use of domain-level background information and event-level management-context information, thereby improving forecasting error metrics in the current setting. However, when the prefix becomes too long, the additionally introduced information may become redundant and may instead interfere with the model’s learning of the main temporal variations.

From the perspective of its specific role, when k = 0, the model does not contain any explicit semantic prefix and, therefore, relies mainly on the numerical sequence itself for forecasting, resulting in relatively insufficient representation of management events and scenario transitions. As k increases moderately, the prefix information can provide useful background conditions for time-series modeling, helping reduce representation confusion between routine context-related fluctuations and other variation patterns, thereby improving long-range forecasting performance. More broadly, the results show that a moderate prefix length performs better overall than either excessively short or excessively long settings, indicating that the role of DKPE is not simply to increase contextual information but, rather, to provide an appropriate amount of task-relevant background information for forecasting. In the current task, k = 8 achieved a favorable balance between information expressiveness and model complexity, and it was therefore adopted as the default setting in the subsequent experiments.

Overall, the sensitivity results can be used to support hyperparameter selection and model-configuration stability. They do not by themselves establish biological interpretability or semantic superiority; rather, they show how different configuration choices affect forecasting performance under the defined experimental protocol.

### 4.7. Exploratory Analyses Supporting Result Interpretation

To avoid mixing exploratory analyses with the main discussion, two supplementary analyses are presented at the end of the Results section. The first examines possible lagged statistical associations between barn environmental variation and active duration, and the second qualitatively visualizes latent representations before the forecasting head. These analyses are intended to support interpretation of the forecasting results, rather than to provide direct evidence of physiological mechanisms, biological-state discrimination, or semantic superiority.

#### 4.7.1. Exploratory Lag-Correlation Analysis

**Figure 8:**
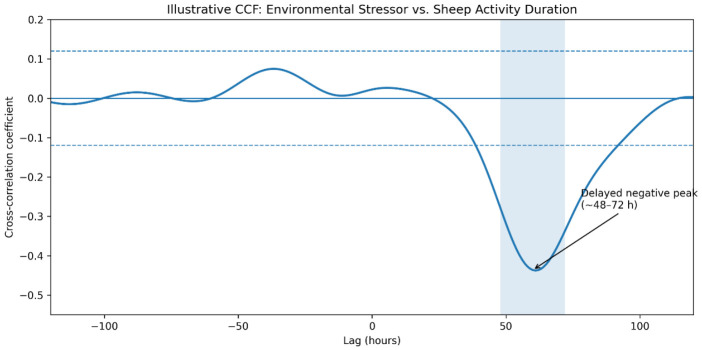
Exploratory cross-correlation analysis between the composite environmental-variation index and active duration in meat sheep. The environmental-variation index was constructed from hourly standardized temperature, relative humidity, NH_3_, CO_2_, PM_2.5_, PM_10_, and light intensity. The analysis was used to support interpretation of possible temporal associations and should not be interpreted as causal physiological evidence.

To provide an exploratory statistical view of possible temporal associations between barn environmental variation and meat sheep activity, we used the Cross-Correlation Function (CCF) to analyze the correlation structure between selected environmental variables and active duration, as shown in Figure 8. This analysis was used as supplementary forecasting-level evidence rather than as direct evidence of physiological causality.

To clarify the environmental variables used in this analysis, the CCF was computed using the hourly aligned environmental variables included in the model input, namely, temperature, relative humidity, NH_3_, CO_2_, PM_2.5_, PM_10_, and light intensity. Before CCF calculation, these variables were processed using the same preprocessing pipeline as the forecasting model: high-frequency environmental measurements were aggregated to hourly statistics, short missing segments were locally corrected according to the rules in Section 2.4, time windows with missing durations longer than 1 h were removed, and continuous variables were standardized using the training-set statistics of the corresponding experimental split.

Because these variables have different units and physical meanings, they were not directly summed in their original scale. Instead, after standardization, a composite environmental-variation index was constructed to represent the overall magnitude of barn environmental fluctuation:

(30) $$E_{t}=\frac{1}{K}\sum_{k=1}^{K}\left|x_{k,t}^{*}\right|,\;K=7,$$

where $x_{k,t}^{*}$ denotes the standardized value of the *k*-th environmental variable at hour *t*, and K=7 corresponds to the seven environmental variables listed above. Active duration was also standardized before the correlation calculation. The CCF was then calculated between the composite environmental-variation index $E_{t}$ and the standardized active duration series $a_{t}^{*}$:

(31) $$\rho \left(\ell \right)=\mathrm{corr}\left(E_{t},a_{t+\ell }^{*}\right),$$

where *ℓ* denotes the lag in hours. A positive lag indicates that environmental variation precedes the change in active duration. The term “environmental variation” is used here only as an exploratory statistical descriptor and should not be interpreted as a clinically or physiologically validated stress index.

As shown in Figure 8, the largest absolute correlation between the composite environmental-variation index and active duration did not occur at the zero-lag position but was mainly observed within the lag interval of approximately 48–72 h, with a peak around 60 h. This pattern suggests a possible delayed statistical association between barn environmental variation and subsequent changes in active duration in this dataset.

Several alternative explanations should also be considered when interpreting the 48–72 h lag. First, both environmental variables and active duration may contain autocorrelation, and such serial dependence can influence the location and magnitude of cross-correlation peaks. Second, routine farm-management activities, such as feeding, manure cleaning, ventilation adjustment, and heating-device operation, may follow repeated daily or cross-day schedules, which can introduce periodic structures into both environmental and animal-state time series. Third, repeated day–night routines and delayed operational adjustments may produce apparent lagged associations even when a single physiological response pathway is not directly verified. Therefore, the observed 48–72 h pattern should be interpreted as an exploratory cross-day statistical association that may reflect the combined effects of environmental dynamics, management cycles, daily routines, and time-series autocorrelation.

However, the CCF result should not be interpreted as evidence of a causal physiological pathway. Rather, it provides exploratory support for using cross-day historical information in the forecasting model, which is consistent with the lookback window results reported above. Further controlled experiments or intervention-based studies would be required to distinguish physiological response delays from management-cycle and autocorrelation effects.

#### 4.7.2. Exploratory Latent-Representation Visualization

**Figure 9:**
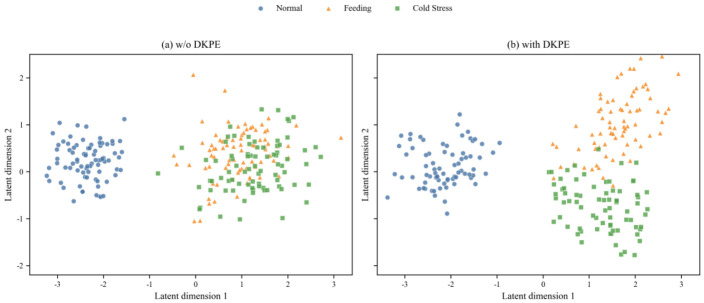
Exploratory t-SNE visualization of hidden representations before the forecasting head, with active duration used as the representative target indicator. The visualization was used to qualitatively examine representation organization under different contextual labels and should not be interpreted as direct evidence of biological-state discrimination.

To qualitatively inspect whether hierarchical contextual prefixes changed the organization of learned representations, we extracted the hidden representations before the forecasting head and visualized them using t-SNE, as shown in Figure 9. This visualization was used as an exploratory representation-level analysis, rather than as statistical proof of semantic superiority or biological-state separation.

As shown in Figure 9a, when hierarchical contextual prefixes were removed, the feeding-labeled and cold-stress-labeled samples showed greater overlap in the two-dimensional t-SNE projection. After introducing hierarchical contextual information, the separation among the three labeled sample groups became more visible in the projected latent space. This result suggests that contextual information changes the distribution of learned representations and may help reduce representation overlap between samples collected under different management or environmental contexts. However, this visualization should not be interpreted as direct evidence that the model identifies the underlying biological causes of these patterns.

Overall, these exploratory analyses are consistent with the quantitative forecasting results, but they should be interpreted only as supplementary statistical and representation-level evidence. They support the use of long-context and management-context-aware modeling without establishing specific physiological mechanisms or direct discrimination between normal and stress states.

## 5. Discussion

### 5.1. Joint Modeling of Environmental Exposure, Management Context, and Meat Sheep State Dynamics

The three core modules of GCL-Sheep should be interpreted as complementary parts of an animal-state forecasting workflow rather than as independent algorithmic additions. The practical difficulty is that future physiological and behavioral indicators may be associated with environmental exposure, animal-history information, management context, and delayed temporal patterns in the monitoring data. CGC, DKPE, and LCPB therefore correspond to three farm-facing modeling requirements: representing environment–animal-state associations, incorporating management background, and using extended historical information for multi-step forecasting.

This interpretation is consistent with previous studies in precision sheep farming and livestock environmental physiology. Sensor-based sheep-monitoring studies have shown that activity-, feeding-, rumination-, and body-temperature-related variables can provide useful information for welfare and health-state assessment [1,2,4,5,8]. In addition, environmental physiology and livestock-house air-quality studies have reported that thermal conditions, ammonia exposure, and particulate matter are associated with feeding behavior, rumination-related activity, respiratory response, thermoregulation, and other behavioral or physiological changes in sheep or sheep–goat housing systems [6,7,37,38]. Therefore, the present discussion interprets the model components as forecasting-oriented representations of these known monitoring dimensions, rather than as experimental proof of new biological mechanisms.

CGC contributes by reorganizing multi-source monitoring variables according to domain-informed associations that are relevant to animal-state forecasting. If environmental, physiological, and behavioral variables are simply concatenated, the model may learn local correlations but may not sufficiently separate domain-informed variable associations from incidental co-fluctuations in a specific barn or season. By integrating prior topology with data-driven adjacency relationships, CGC helps the model represent associations among barn environment, animal-state indicators, and behavioral outputs in a structured way. This should be understood as a forecasting-level representation strategy, not as direct evidence of causal biological pathways.

DKPE contributes by adding the operational background under which numerical changes occur. In real sheep farms, changes in activity, rumination, feeding duration, or body temperature may occur under different management contexts, such as feeding, manure cleaning, ventilation adjustment, day–night transition, or heating operation. Previous decision-support studies in precision sheep farming have also emphasized that sensor readings should be interpreted together with operational background and expert knowledge, rather than as isolated values [8,9,10,21]. Accordingly, DKPE should be interpreted as a way to provide management-context information to the forecasting model, rather than as evidence that the model fully understands the biological meaning of each behavioral fluctuation.

LCPB contributes by modeling longer temporal contexts in which environmental variation, management events, and animal-state indicators may show delayed statistical associations. A short-window model is more likely to focus on local fluctuations, whereas long-context modeling can include day–night rhythms, repeated management events, and cross-day environmental variation. Because LCPB receives representations already informed by variable relationships and management context, it may better capture delayed temporal associations between environmental variation and future animal-state indicators. This interpretation is consistent with the lookback-window and exploratory lag-correlation analyses reported in the Results section, but it should not be interpreted as direct proof of a specific physiological lag mechanism.

The ablation results provide forecasting-level support for this interpretation. Removing CGC reduced the benefit of graph-based variable representation, removing DKPE reduced the benefit of management-context information, and removing the key long-context design in LCPB weakened the model’s ability to use extended historical information. These degradations indicate that the performance gain is associated with the coordinated use of structural, contextual, and temporal information for the same animal-state forecasting task, rather than with a single component alone.

In practical terms, GCL-Sheep converts heterogeneous farm monitoring records into forecasts that are more closely aligned with animal-state monitoring needs. The model not only extrapolates a target sequence but also uses environmental measurements, management background, and cross-day temporal information to support context-aware forecasting of future sheep behavior and physiological indicators. The discussion of these components is therefore intended to explain their contribution to forecasting performance and farm decision support, rather than to claim direct validation of biological mechanisms.

### 5.2. Implications for Precision Monitoring and Proactive Management in Meat Sheep Farming

The main practical significance of this study is that it shifts the focus of smart sheep farming from monitoring barn conditions alone toward forecasting future animal-state indicators. Environmental variables such as temperature, humidity, ammonia concentration, carbon dioxide concentration, and particulate matter describe barn exposure conditions, whereas active duration, rumination duration, feeding duration, intense exercise duration, and body temperature describe animal-state responses that are more directly connected to welfare monitoring and management needs. This application-oriented interpretation is consistent with previous reviews and decision-support studies in precision sheep farming and precision livestock farming [8,9,10,21].

The model outputs should therefore be interpreted as decision-support information rather than as direct diagnostic conclusions. By jointly using environmental measurements, individual historical states, and management events, GCL-Sheep provides future estimates that may help identify abnormal trends, potential health-risk signals, or management periods requiring attention. For farm use, such forecasts may support prioritization of ventilation adjustment, thermal regulation, feeding arrangement, inspection scheduling, and targeted observation. However, these outputs should complement, rather than replace, direct animal observation, veterinary judgment, and established welfare assessment protocols.

The multi-step nature of the forecasts is potentially relevant for proactive management. The forecasting results and exploratory lag-correlation analysis suggest that animal-state indicators may contain useful delayed temporal information under environmental and management changes. When the model maintains performance at longer forecasting horizons, it may provide a useful management window for earlier inspection, environmental adjustment, or targeted observation. However, this should be interpreted as retrospective forecasting evidence rather than as validation of a real-time early-warning or intervention system. Further field validation is still needed before defining practical intervention thresholds.

The LODO cross-domain and few-shot adaptation results further suggest deployment potential in new farming scenarios. Actual sheep farms differ in climate, barn structure, sensor configuration, stocking density, and management rhythm, so a model that performs well only in one scenario may have limited practical value. In this study, GCL-Sheep maintained useful transfer performance across unseen farm–season–barn domains, and its performance improved after introducing a small amount of target-domain data. This indicates that the learned variable-association and management-context representations may support low-cost adaptation when the model is introduced into a new farm. Nevertheless, the present evaluation was conducted retrospectively using historical monitoring data, and no real-time system deployment, online model updating, farmer-facing alert interface, or farmer intervention trial was conducted in this study. Therefore, broader multi-region validation and prospective deployment tests are still required.

Several practical issues still need attention before routine deployment. Sensor failures, missing records, changes in management workflow, differences in stocking density, and farm-specific decision thresholds may affect how forecasts are used in practice. Future on-farm validation should therefore connect model outputs with explicit early-warning thresholds, welfare assessment protocols, farmer feedback, and intervention records. Such validation would help determine how animal-state forecasts can be integrated into daily farm management, how alerts should be presented to farmers, and how much human oversight is required for reliable decision support.

## 6. Conclusions

This study developed GCL-Sheep, a management-context-aware forecasting model for predicting key physiological and behavioral indicators in Hu sheep using multi-source farm monitoring data. By integrating barn environmental measurements, historical animal-state records, and management-context information, the model provides multi-step forecasts of active duration, rumination duration, feeding duration, intense exercise duration, and body temperature. The purpose of the framework is to provide forward-looking animal-state information for management-oriented decision support in precision sheep farming, rather than to directly diagnose welfare or health status.

The main evidence shows that GCL-Sheep achieved promising retrospective forecasting performance compared with XGBoost, LSTM, TCN, Transformer, PatchTST, and Time-LLM under the in-domain setting, with clearer advantages at longer forecasting horizons. For active-duration forecasting at H=12, the model reduced the MAE and RMSE by 20.0% and 19.2%, respectively, compared with the second-best baseline, and it improved $R^{2}$ by 0.079. The lookback-window experiment indicated that a 96 h historical window provided a favorable balance between prediction accuracy, temporal coverage, and computational cost. In addition, the Leave-One-Domain-Out and few-shot target-domain fine-tuning results suggest that the model has potential for cross-scenario forecasting and limited-data adaptation under new farm–season–barn conditions.

Overall, this study contributes an applied AI workflow for transforming heterogeneous farm monitoring records into sensor-derived animal-state forecasts. The results support the value of jointly using environmental exposure, historical animal-state information, and management context for multi-step forecasting in precision sheep farming. However, the present study evaluated retrospective predictive accuracy only. It did not validate welfare-threshold-based early warning, clinical diagnosis, real-time deployment, or farmer intervention outcomes. Therefore, the model outputs should be regarded as decision-support information that may assist in inspection scheduling, environmental adjustment, and targeted observation, rather than as direct diagnostic or welfare-intervention conclusions.

Several limitations remain. First, the number and diversity of available farm–season–barn domains are still limited, and broader external validation across independent farms, regions, seasons, breeds, and management systems is needed. Second, several analyses used active duration as the representative target indicator, while other physiological and behavioral indicators may differ in noise level, temporal regularity, event sparsity, and forecasting difficulty. Third, the full raw farm-monitoring data have limited accessibility because they contain farm operation records and individual animal monitoring information. To support reproducibility, future work should provide code, model configurations, preprocessing scripts, trained-model settings, and anonymized or representative sample-generation templates where permitted by data-sharing agreements. Finally, practical deployment still requires prospective field trials with explicit early-warning thresholds, welfare assessment protocols, farmer feedback, and intervention records. Future work should therefore focus on larger multi-region datasets, individual-difference analysis, more fine-grained multi-indicator forecasting, interpretable decision rules, and real-farm deployment tests under routine management conditions.

## Figures and Tables

**Table 1:** Biological, environmental, and practical relevance of the monitored variables.

Variable Group	Monitored Variables	Biological or Environmental Meaning	Practical Management Relevance
Environmental exposure	Temperature and relative humidity	Thermal environment and potential heat/cold stress background	Heating, ventilation, thermal regulation, and comfort management
Environmental exposure	NH_3_ and CO_2_	Barn air-quality exposure and ventilation-related accumulation	Ventilation adjustment, manure cleaning, and air-quality risk screening
Environmental exposure	PM_2.5_ and PM_10_	Particulate exposure and dust conditions in the barn	Dust-control management, inspection scheduling, and environmental improvement
Environmental exposure	Light intensity	Lighting background and day–night rhythm information	Activity-rhythm interpretation and routine management scheduling
Animal-state response	Active duration	General activity allocation and movement level	Welfare monitoring, abnormal activity screening, and inspection prioritization
Animal-state response	Rumination duration	Ingestive and digestive behavioral state	Feeding management, health-risk warning, and behavioral assessment
Animal-state response	Feeding duration	Feed-intake-related behavioral allocation	Feeding management and production-state monitoring
Animal-state response	Intense exercise duration	Abrupt or high-intensity movement events	Abnormal movement detection and stress-related warning support
Animal-state response	Body temperature	Thermoregulatory and physiological state	Health-risk warning, thermal-stress screening, and targeted intervention
Management context	Day–night status, feeding or fasting stage, manure cleaning, vaccination, and equipment operation	Operational background for interpreting animal-state changes	Context-aware interpretation of behavioral and physiological fluctuations under real production conditions

**Table 2:** Approximate record retention and supervised sample generation after domain screening and preprocessing.

Domain	Raw Hourly Records	After QC	Missing Processed	Aligned Usable	Supervised Samples
D1	∼118,000	∼116,000	∼113,000	∼111,000	∼105,000
D2	∼78,000	∼76,000	∼74,000	∼72,000	∼68,000
D3	∼83,000	∼81,000	∼79,000	∼77,000	∼73,000
D4	∼34,000	∼33,000	∼32,000	∼31,000	∼28,000
D5	∼36,000	∼35,000	∼34,000	∼33,000	∼30,000
D1–D5 total	∼349,000	∼342,000	∼332,000	∼325,000	∼304,000
Dataset B summer/autumn not assigned	∼121,000	–	–	–	–
Original total	∼470,000	–	–	–	–

**Table 3:** Summary of data sources, domain assignment, and modeling sample size.

Dataset/ Domain	Site/Barn	Period Used	No. of Animals	Usable Hourly Records	Generated Samples	Domain Assignment and Notes
Dataset A/D1	Daqing, Barn #1	June 2024–October 2024	40	∼111,000	∼105,000	Daqing summer domain; hot and humid conditions with strong ventilation demand.
Dataset B/D2	Daqing, Barn #2	Winter segment	40	∼72,000	∼68,000	Daqing winter domain; retained after quality control and temporal alignment.
Dataset B/D3	Daqing, Barn #2	Spring segment	40	∼77,000	∼73,000	Daqing spring domain; retained after quality control and temporal alignment.
Dataset B/Not assigned	Daqing, Barn #2	Summer/autumn portions	40	–	–	Not used as an independent modeling domain. ^a^
Dataset C/D4	Aksu, Barn #3	Autumn segment	35	∼31,000	∼28,000	Aksu autumn domain; dry conditions with large diurnal temperature variation.
Dataset C/D5	Aksu, Barn #3	Winter segment	35	∼33,000	∼30,000	Aksu winter domain; enclosed barn condition with heating and ventilation demand.
Total/D1–D5	–	–	115	∼325,000	∼304,000	Final modeling domains used for in-domain forecasting, LODO cross-domain evaluation, and few-shot adaptation.

^a^ The summer/autumn portions of Dataset B were not assigned to independent modeling domains because they overlapped with production-batch transition, monitoring-system adjustment, and changes in continuously tracked ear-tag IDs, resulting in insufficient continuity of synchronized individual-state records after quality control.

**Table 4:** Approximate missingness rate of main variables before imputation or window removal.

Variable	D1	D2	D3	D4	D5	Main Handling Rule
Temperature	∼0.8%	∼1.0%	∼0.9%	∼1.2%	∼1.1%	Short gaps interpolated; long gaps removed.
Relative humidity	∼0.9%	∼1.1%	∼1.0%	∼1.3%	∼1.2%	Short gaps interpolated; long gaps removed.
NH_3_	∼2.1%	∼2.6%	∼2.4%	∼3.8%	∼3.5%	Interpolation or forward filling for short gaps; long gaps removed.
CO_2_	∼1.8%	∼2.3%	∼2.1%	∼3.2%	∼3.0%	Interpolation or forward filling for short gaps; long gaps removed.
PM_2.5_	∼2.9%	∼3.4%	∼3.1%	∼5.6%	∼5.1%	Short gaps corrected; long dust-related gaps removed.
PM_10_	∼3.2%	∼3.7%	∼3.4%	∼5.9%	∼5.4%	Short gaps corrected; long dust-related gaps removed.
Light intensity	∼0.7%	∼0.8%	∼0.8%	∼1.0%	∼1.0%	Short gaps interpolated; long gaps removed.
Active duration	∼1.4%	∼1.7%	∼1.6%	∼2.2%	∼2.0%	Incomplete animal-state sequences screened.
Rumination duration	∼1.6%	∼1.9%	∼1.8%	∼2.5%	∼2.3%	Incomplete animal-state sequences screened.
Feeding duration	∼1.5%	∼1.8%	∼1.7%	∼2.4%	∼2.2%	Incomplete animal-state sequences screened.
Intense exercise duration	∼1.7%	∼2.0%	∼1.9%	∼2.6%	∼2.4%	Incomplete animal-state sequences screened.
Body temperature	∼1.3%	∼1.6%	∼1.5%	∼2.1%	∼2.0%	Incomplete or abnormal sequences screened.
Management context	<1.0%	<1.0%	<1.0%	∼1.2%	∼1.1%	Recovered using logs, equipment states, or time-based rules; otherwise marked as unknown.

**Table 5:** Example of a preprocessed single-time-step feature sample.

Timestamp	Sheep ID	Environmental Features	Physiological/Behavioral Features	Management Context Features
25 September 2025 08:00	01	Ambient ${\mathrm{Temp}}_{\mathrm{mean}}$, ${\mathrm{RH}}_{\mathrm{mean}}$, ${\mathrm{NH}}_{3,\mathrm{mean}}$, ${\mathrm{CO}}_{2,\mathrm{mean}}$, ${\mathrm{PM}}_{2.5,\mathrm{mean}}$, ${\mathrm{Light}}_{\mathrm{mean}}$, …	Active Duration, Rumination Duration, Feeding Duration, Intense Exercise Duration, Body Temperature, …	Day/Night, Feeding/Fasting, Cleaning, Vaccination, Fan On/Off, Cooling Pad On/Off, …
25 September 2025 09:00	01	Ambient ${\mathrm{Temp}}_{\mathrm{mean}}$, ${\mathrm{RH}}_{\mathrm{mean}}$, ${\mathrm{NH}}_{3,\mathrm{mean}}$, ${\mathrm{CO}}_{2,\mathrm{mean}}$, ${\mathrm{PM}}_{2.5,\mathrm{mean}}$, ${\mathrm{Light}}_{\mathrm{mean}}$, …	Active Duration, Rumination Duration, Feeding Duration, Intense Exercise Duration, Body Temperature, …	Day/Night, Feeding/Fasting, Cleaning, Vaccination, Fan On/Off, Cooling Pad On/Off, …
25 September 2025 10:00	01	Ambient ${\mathrm{Temp}}_{\mathrm{mean}}$, ${\mathrm{RH}}_{\mathrm{mean}}$, ${\mathrm{NH}}_{3,\mathrm{mean}}$, ${\mathrm{CO}}_{2,\mathrm{mean}}$, ${\mathrm{PM}}_{2.5,\mathrm{mean}}$, ${\mathrm{Light}}_{\mathrm{mean}}$, …	Active Duration, Rumination Duration, Feeding Duration, Intense Exercise Duration, Body Temperature, …	Day/Night, Feeding/Fasting, Cleaning, Vaccination, Fan On/Off, Cooling Pad On/Off, …
…	…	…	…	…
28 December 2025 21:00	35	Ambient ${\mathrm{Temp}}_{\mathrm{mean}}$, ${\mathrm{RH}}_{\mathrm{mean}}$, ${\mathrm{NH}}_{3,\mathrm{mean}}$, ${\mathrm{CO}}_{2,\mathrm{mean}}$, ${\mathrm{PM}}_{2.5,\mathrm{mean}}$, ${\mathrm{Light}}_{\mathrm{mean}}$, …	Active Duration, Rumination Duration, Feeding Duration, Intense Exercise Duration, Body Temperature, …	Day/Night, Feeding/Fasting, Cleaning, Vaccination, Fan On/Off, Cooling Pad On/Off, …

**Table 6:** Baseline implementation settings for fair comparison.

Model	Input Features	Lookback Setting	Main Hyperparameters	Training Protocol
XGBoost	Same environmental, historical animal-state, and management-context features as GCL-Sheep; historical window flattened into a vector	L=96 h flattened window	500 estimators, maximum depth 6, learning rate 0.05, subsample 0.8, column-sample ratio 0.8	Validation-set early stopping; same target-specific forecasting tasks and splits as GCL-Sheep
LSTM	Same multi-source hourly feature sequence as GCL-Sheep	L=96 h sequence	Two recurrent layers, hidden dimension 128, dropout 0.1	Adam optimizer, learning rate $1\times 10^{-3}$, batch size 64, early stopping on validation loss
TCN	Same multi-source hourly feature sequence as GCL-Sheep	L=96 h sequence	Four temporal convolution blocks, hidden dimension 128, kernel size 3, dilation factors 1–8, dropout 0.1	Adam optimizer, learning rate $1\times 10^{-3}$, batch size 64, early stopping on validation loss
Transformer	Same multi-source hourly feature sequence as GCL-Sheep	L=96 h sequence	Two encoder layers, hidden dimension 128, four attention heads, feed-forward dimension 256, dropout 0.1	Adam optimizer, learning rate $1\times 10^{-3}$, batch size 64, early stopping on validation loss
PatchTST	Same multi-source hourly feature sequence as GCL-Sheep	L=96 h sequence; patch length 16 h; stride 8 h	Two encoder layers, hidden dimension 128, four attention heads, feed-forward dimension 256, dropout 0.1	Adam optimizer, learning rate $1\times 10^{-3}$, batch size 64, early stopping on validation loss
Time-LLM	Same multi-source hourly feature sequence as GCL-Sheep; time-series features reprogrammed into prompt-like representations	L=96 h sequence	Frozen pretrained language-model backbone; trainable reprogramming and forecasting layers; hidden dimension 128; dropout 0.1	Same chronological splits and target-specific forecasting tasks; trainable layers optimized using validation loss

**Table 7:** Approximate number of trainable parameters and repeated-run settings.

Model	Trainable Parameters	Repeated-Run Setting
XGBoost	N.A. (tree-based)	Seeds 41, 42, 43
LSTM	∼0.47 M	Seeds 41, 42, 43
TCN	∼0.39 M	Seeds 41, 42, 43
Transformer	∼0.56 M	Seeds 41, 42, 43
PatchTST	∼0.61 M	Seeds 41, 42, 43
Time-LLM	∼0.84 M trainable; pretrained backbone frozen	Seeds 41, 42, 43
GCL-Sheep	∼0.92 M	Seeds 41, 42, 43

**Table 8:** Key implementation settings and hyperparameter-selection rationale.

Component	Setting	Selection Rationale
Graph fusion	α=0.5	Selected from {0,0.25,0.5,0.75,1.0} according to validation performance and sensitivity analysis; balances prior-knowledge constraints and data-driven adjacency learning.
GCN depth	Two GCN layers	Provides cross-variable propagation while avoiding excessive depth and potential over-smoothing under the current sample size.
Hidden dimension	128	Chosen to balance representation capacity, computational cost, and validation stability across forecasting tasks.
Dropout	0.1	Used as mild regularization to reduce overfitting while preserving temporal and graph-structured information.
Temporal patching	Patch length = 16 h; stride = 8 h	Reduces the effective sequence length for long-context modeling while retaining local temporal continuity and short-term fluctuations.
Alignment loss	KL divergence	Used to align the latent distributions of graph-enhanced numerical features and hierarchical semantic prefixes in a differentiable manner.
Loss weights	$\lambda_{1}=1.0$, $\lambda_{2}=0.1$	Keeps the forecasting loss as the dominant objective and uses the alignment loss as an auxiliary regularization term.
Temperature	τ=1.0	Selected after preliminary validation to avoid excessive smoothing or sharpening of latent distributions.
Prefix length	k=8	Selected according to validation performance and sensitivity analysis; balances contextual information capacity and model complexity.

**Table 9:** Performance comparison of all models under different forecasting horizons *H*.

Model	Metric	H=1	H=3	H=6	H=12
GCL-Sheep	MAE	0.105±0.002	0.128±0.004	0.154±0.003	0.188±0.006
	RMSE	0.142±0.003	0.175±0.005	0.212±0.005	0.265±0.007
	$R^{2}$	0.962±0.002	0.925±0.003	0.884±0.006	0.821±0.008
Time-LLM	MAE	0.118±0.004	0.149±0.007	0.184±0.006	0.235±0.011
	RMSE	0.165±0.006	0.206±0.008	0.279±0.010	0.328±0.014
	$R^{2}$	0.945±0.004	0.892±0.007	0.836±0.008	0.742±0.013
PatchTST	MAE	0.122±0.003	0.147±0.006	0.191±0.009	0.252±0.012
	RMSE	0.170±0.005	0.204±0.007	0.268±0.011	0.355±0.016
	$R^{2}$	0.938±0.004	0.900±0.006	0.823±0.011	0.715±0.015
Transformer	MAE	0.135±0.006	0.176±0.007	0.229±0.011	0.310±0.018
	RMSE	0.188±0.008	0.246±0.009	0.299±0.014	0.425±0.023
	$R^{2}$	0.912±0.006	0.841±0.010	0.749±0.014	0.605±0.022
TCN	MAE	0.142±0.005	0.173±0.009	0.241±0.010	0.335±0.021
	RMSE	0.195±0.007	0.235±0.012	0.346±0.018	0.460±0.025
	$R^{2}$	0.901±0.008	0.847±0.009	0.732±0.017	0.552±0.028
LSTM	MAE	0.148±0.006	0.202±0.012	0.276±0.016	0.385±0.024
	RMSE	0.205±0.009	0.284±0.015	0.384±0.021	0.525±0.030
	$R^{2}$	0.890±0.008	0.788±0.015	0.649±0.024	0.425±0.034
XGBoost	MAE	0.150±0.003	0.196±0.008	0.284±0.014	0.390±0.020
	RMSE	0.210±0.006	0.276±0.012	0.412±0.023	0.540±0.028
	$R^{2}$	0.885±0.006	0.798±0.011	0.638±0.020	0.412±0.030

**Table 10:** Performance comparison of different models under the LODO cross-domain forecasting setting (active duration, H=12).

Model	MAE	RMSE	$R^{2}$
GCL-Sheep	0.201±0.007	0.278±0.010	0.792±0.012
Time-LLM	0.245±0.011	0.345±0.016	0.715±0.019
PatchTST	0.268±0.014	0.382±0.019	0.662±0.022
Transformer	0.295±0.016	0.428±0.024	0.584±0.027
TCN	0.322±0.019	0.465±0.025	0.510±0.031
LSTM	0.354±0.023	0.512±0.031	0.435±0.038
XGBoost	0.380±0.017	0.550±0.029	0.392±0.034

**Table 11:** Per-left-out-domain LODO performance of GCL-Sheep for active-duration forecasting at H=12.

Left-Out Domain	Target-Domain Scenario	MAE	RMSE	$R^{2}$
D1	Daqing summer Barn #1	0.194±0.006	0.270±0.009	0.806±0.010
D2	Daqing winter Barn #2	0.207±0.010	0.286±0.014	0.783±0.017
D3	Daqing spring Barn #2	0.198±0.007	0.274±0.011	0.797±0.014
D4	Aksu autumn Barn #3	0.215±0.013	0.296±0.019	0.765±0.024
D5	Aksu winter Barn #3	0.191±0.005	0.264±0.008	0.809±0.009
Average	All LODO rounds	0.201±0.007	0.278±0.010	0.792±0.012

**Table 12:** Few-shot adaptation results of GCL-Sheep under different proportions of target-domain samples (active duration, H=12).

Target-Domain Sample Ratio	MAE	RMSE	$R^{2}$
0% (Zero-shot)	0.201±0.007	0.278±0.010	0.792±0.012
5%	0.192±0.006	0.265±0.009	0.805±0.011
10%	0.185±0.006	0.254±0.007	0.818±0.010
20%	0.178±0.005	0.242±0.008	0.830±0.009

**Table 13:** Effects of different historical lookback window lengths *L* on the performance of GCL-Sheep (H=6).

Look-Back Window *L*	MAE	RMSE	$R^{2}$
24	0.195±0.007	0.268±0.010	0.805±0.012
48	0.178±0.006	0.245±0.007	0.842±0.010
72	0.162±0.004	0.224±0.008	0.868±0.007
96	0.154±0.003	0.212±0.005	0.884±0.006
120	0.161±0.006	0.218±0.006	0.872±0.009

**Table 14:** Cross-variable forecasting performance of GCL-Sheep for the five target indicators under the in-domain setting at H=12.

Target Indicator	Unit	MAE	RMSE	$R^{2}$
Active duration	min h^−1^	3.42±0.12	4.78±0.17	0.821±0.008
Rumination duration	min h^−1^	2.86±0.10	4.06±0.16	0.846±0.007
Feeding duration	min h^−1^	2.54±0.09	3.72±0.11	0.854±0.006
Intense exercise duration	min h^−1^	1.48±0.08	2.11±0.13	0.786±0.012
Body temperature	°C	0.12±0.01	0.17±0.02	0.832±0.009

**Table 15:** Performance comparison of ablation experiments on the core modules.

Model	Description	H=6 MAE	H=6 RMSE	H=6 $R^{2}$	H=12 MAE	H=12 RMSE	H=12 $R^{2}$
GCL-Sheep	Full Model	0.154±0.003	0.212±0.005	0.884±0.006	0.188±0.006	0.265±0.007	0.821±0.008
w/o CGC	Remove graph fusion; use linear feature concatenation	0.178±0.006	0.255±0.010	0.852±0.010	0.224±0.010	0.315±0.013	0.765±0.018
w/o Hierarchical DKPE	Remove hierarchical contextual prefixes; use numerical inputs only	0.168±0.005	0.230±0.009	0.835±0.014	0.235±0.012	0.318±0.014	0.720±0.021
w/o Patching	Remove the patching mechanism in LCPB	0.172±0.007	0.235±0.008	0.850±0.011	0.215±0.009	0.298±0.015	0.772±0.016

**Table 16:** Input-source ablation results under the in-domain forecasting setting.

Variant	Removed Input Source	H=6 MAE	H=6 RMSE	H=6 $R^{2}$	H=12 MAE	H=12 RMSE	H=12 $R^{2}$
Full model	None	0.154±0.003	0.212±0.005	0.884±0.006	0.188±0.006	0.265±0.007	0.821±0.008
w/o environmental variables	Temperature, relative humidity, NH_3_, CO_2_, PM_2.5_, PM_10_, and light intensity	0.182±0.007	0.252±0.011	0.842±0.012	0.231±0.011	0.323±0.016	0.741±0.020
w/o management-context variables	Day–night status, feeding/fasting stage, manure cleaning, vaccination, and equipment-operation states	0.169±0.006	0.232±0.009	0.861±0.009	0.218±0.010	0.302±0.013	0.765±0.016
w/o animal-history variables	Historical physiological and behavioral indicators in the input window	0.210±0.012	0.292±0.016	0.780±0.018	0.268±0.017	0.371±0.023	0.657±0.030

**Table 17:** Effect of the graph fusion weight α on activity-duration forecasting performance (L=96, H=12).

α	MAE	RMSE	$R^{2}$
0.00	0.214±0.010	0.295±0.014	0.768±0.017
0.25	0.201±0.008	0.281±0.012	0.793±0.013
0.50	0.188±0.006	0.265±0.007	0.821±0.008
0.75	0.194±0.005	0.273±0.009	0.808±0.011
1.00	0.207±0.011	0.287±0.013	0.784±0.016

**Table 18:** Effects of prefix length *k* on activity-duration forecasting performance (L=96, H=12).

*k*	MAE	RMSE	$R^{2}$
0	0.203±0.009	0.284±0.011	0.790±0.014
4	0.194±0.006	0.273±0.010	0.808±0.009
8	0.188±0.006	0.265±0.007	0.821±0.008
12	0.191±0.007	0.269±0.009	0.815±0.012
16	0.197±0.008	0.277±0.013	0.801±0.015

## Data Availability

The raw data supporting the conclusions of this article will be made available by the authors on request.

## Appendix A. Prior Edges Used for Static Prior Graph Initialization

This appendix lists the complete prior edges used to initialize the static prior adjacency matrix $A_{\mathrm{prior}}$. For readability, the prior edges are organized into four tables according to their functional type: air-quality-related cross-modal edges, thermal-related cross-modal edges, particulate/light-related cross-modal edges, and intra-physiological/intra-environmental edges.

**Table A1:** Air-quality-related cross-modal prior edges used to initialize $A_{\mathrm{prior}}$.

Source Node	Target Node	Edge Type	Biological or Environmental Justification	Reference Basis
NH_3_	Rumination duration	Directed	Ammonia exposure may affect ingestive behavior, chewing activity during rumination, respiratory response, and stress-related physiological indicators.	[37]
NH_3_	Feeding duration	Directed	Ammonia exposure may affect feed intake and feeding-related behavior.	[37]
CO_2_	Rumination duration	Directed	CO_2_ was treated as a ventilation and air-quality indicator that may be associated with ingestive-behavior changes under barn air-quality variation.	[37,38]
CO_2_	Feeding duration	Directed	CO_2_ was used as an air-quality and ventilation-related variable that may reflect environmental background relevant to feeding behavior.	[37,38]

**Table A2:** Thermal-related cross-modal prior edges used to initialize $A_{\mathrm{prior}}$.

Source Node	Target Node	Edge Type	Biological or Environmental Justification	Reference Basis
Temperature	Active duration	Directed	Thermal conditions may influence activity allocation and behavioral responses in sheep.	[6,7]
Temperature	Intense exercise duration	Directed	Heat or cold background may be associated with changes in high-intensity movement and activity patterns.	[6,7]
Temperature	Body temperature	Directed	Ambient temperature is related to thermoregulatory status and body-temperature variation.	[6,7]
Relative humidity	Active duration	Directed	Relative humidity contributes to the thermal environment and may be associated with activity allocation.	[6,7]
Relative humidity	Intense exercise duration	Directed	Humidity may interact with thermal conditions and affect activity-related behavior.	[6,7]
Relative humidity	Body temperature	Directed	Humidity is part of the thermal-stress background and may be associated with thermoregulatory responses.	[6,7]

**Table A3:** Particulate- and light-related cross-modal prior edges used to initialize $A_{\mathrm{prior}}$.

Source Node	Target Node	Edge Type	Biological or Environmental Justification	Reference Basis
PM_2.5_	Active duration	Directed	Fine particulate exposure reflects barn air-quality and dust conditions that may be associated with behavioral-state changes.	[38]
PM_10_	Active duration	Directed	Coarse particulate exposure reflects dust load and barn air-quality variation that may influence animal activity patterns.	[38]
PM_2.5_	Body temperature	Directed	Particulate exposure was treated as an air-quality background variable potentially related to physiological-state variation.	[38]
PM_10_	Body temperature	Directed	PM_10_ was included as a particulate-exposure node reflecting barn air-quality conditions relevant to physiological monitoring.	[38]
Light intensity	Active duration	Directed	Light background and day–night rhythm may be associated with movement and activity allocation.	[8,10]
Light intensity	Feeding duration	Directed	Light condition and daily rhythm may affect feeding-related activity under routine management.	[8,10]

**Table A4:** Intra-physiological and intra-environmental prior edges used to initialize $A_{\mathrm{prior}}$.

Source Node	Target Node	Edge Type	Biological or Environmental Justification	Reference Basis
Rumination duration	Feeding duration	Undirected	Rumination and feeding are closely related ingestive-behavior indicators and may show coordinated or alternating time allocation.	[1,4,5]
Intense exercise duration	Body temperature	Undirected	High-intensity movement may be accompanied by short-term thermoregulatory variation.	[6,7]
Active duration	Intense exercise duration	Undirected	General activity and high-intensity movement are related movement-state indicators.	[1,4]
Temperature	Relative humidity	Undirected	Temperature and relative humidity often covary under ventilation and heating conditions.	[6,7]
PM_2.5_	PM_10_	Undirected	PM_2.5_ and PM_10_ are related particulate-matter indicators and may fluctuate together under dust exposure.	[38]
NH_3_	CO_2_	Undirected	NH_3_ and CO_2_ were both treated as air-quality and ventilation-related indicators in barn environments.	[38]

## References

[1] do Nascimento Amorim M., Turco S.H.N., dos Santos Costa D., Ferreira I.J.S., da Silva W.P., Sabino A.L.C., da Silva-Miranda K.O. (2024). Discrimination of ingestive behavior in sheep using an electronic device based on a triaxial accelerometer and machine learning. Comput. Electron. Agric..

[2] Suhaimi A.F., Mutiara G.A., Rizal M.F. (2025). Leveraging Embedded Accelerometers and Machine Learning for Real-Time Sheep Behavior Classification in Precision Farming. IEEE Access.

[3] Jin Z., Shu H., Hu T., Jiang C., Yan R., Qi J., Wang W., Guo L. (2024). Behavior classification and spatiotemporal analysis of grazing sheep using deep learning. Comput. Electron. Agric..

[4] Zhang M., Zhu Y., Wu J., Zhao Q., Zhang X., Luo H. (2024). Improved composite deep learning and multi-scale signal features fusion enable intelligent and precise behaviors recognition of fattening Hu sheep. Comput. Electron. Agric..

[5] Schneidewind S.J., Al Merestani M.R., Schmidt S., Schmidt T., Thöne-Reineke C., Wiegard M. (2023). Rumination Detection in Sheep: A Systematic Review of Sensor-Based Approaches. Animals.

[6] Li F., Yang Y., Jenna K., Xia C., Lv S., Wei W. (2018). Effect of heat stress on the behavioral and physiological patterns of Small-tail Han sheep housed indoors. Trop. Anim. Health Prod..

[7] Ferreira J., Crisóstomo C., Marques N.M., Bernardi R.F., de Sousa Corrêa L., Gonçalves L.F., de Sousa Otávio K., Abdalla A.L., da Costa R.L.D. (2025). Effects of heat stress on feeding and drinking behavior of confined Texel sheep in a tropical environment. Appl. Anim. Behav. Sci..

[8] Wang Y., Wang X., Liu K., Cuan K., Hua Z., Li K., Wang K. (2025). Non-invasive monitoring for precision sheep farming: Development, challenges, and future perspectives. Comput. Electron. Agric..

[9] Villeneuve E., Abi Akle A., Merlo C., Masson D., Terrasson G., Llaria A. (2019). Decision support in precision sheep farming. IFAC PapersOnLine.

[10] Noor A., Corke M.J., Tovar E. (2023). Sheep health behavior analysis in machine learning: A short comprehensive survey. Smart Agric. Technol..

[11] Himel G.M.S., Islam M.M., Rahaman M. (2024). Vision intelligence for smart sheep farming: Applying ensemble learning to detect sheep breeds. Artif. Intell. Agric..

[12] Yu R., Wei X., Liu Y., Yang F., Shen W., Gu Z. (2024). Research on automatic recognition of dairy cow daily behaviors based on deep learning. Animals.

[13] Hyndman R.J., Khandakar Y. (2008). Automatic time series forecasting: The forecast package for R. J. Stat. Softw..

[14] Hyndman R.J., Koehler A.B., Snyder R.D., Grose S. (2002). A state space framework for automatic forecasting using exponential smoothing methods. Int. J. Forecast..

[15] Nascimento S.T., da Silva I.J.O., Maia A.S.C., de Castro A.C., Vieira F.M.C. (2014). Mean surface temperature prediction models for broiler chickens—A study of sensible heat flow. Int. J. Biometeorol..

[16] Barbosa L.V., Lima N.D.d.S., Barros J.d.S.G., de Moura D.J., Estellés F., Ramón-Moragues A., Calvet-Sanz S., García A.V. (2024). Predicting risk of ammonia exposure in broiler housing: Correlation with incidence of health issues. Animals.

[17] Feng D., Zhou B., Hassan S.G., Xu L., Liu T., Cao L., Liu S., Guo J. (2022). A hybrid model for temperature prediction in a sheep house. Animals.

[18] Huang J., Guo J., Wu H., Zhang X., Liu S., Hassan S.G. (2022). Short-Term Prediction of Ammonia Levels in Geese Houses Based on Combined Feature Selector and Random Forest. Res. Sq..

[19] García R., Aguilar J., Toro M., Pinto A., Rodríguez P. (2020). A systematic literature review on the use of machine learning in precision livestock farming. Comput. Electron. Agric..

[20] Li Z.L., Zhang G.W., Yu J., Xu L.Y. (2023). Dynamic graph structure learning for multivariate time series forecasting. Pattern Recognit..

[21] Nikolopoulou M.P., Gelasakis A.I., Demestichas K., Kalogianni A.I., Papada I., Lamprou P.A., Chalkos A., Manavis E., Bartzanas T. (2026). Towards Decision Support in Precision Sheep Farming: A Data-Driven Approach Using Multimodal Sensor Data. Ruminants.

[22] Guo Z., Yin Z., Lyu Y., Wang Y., Chen S., Li Y., Zhang W., Gao P. (2024). Research on indoor environment prediction of pig house based on OTDBO–TCN–GRU algorithm. Animals.

[23] Musabimana J., Xie Q., Zhou H., Zheng P., Liu H., Ma T., Liu J. (2025). A Lightweight Hybrid Model for Accurate Ammonia Prediction in Pig Houses. Smart Agric. Technol..

[24] Lin D., Kenéz Á., McArt J.A., Li J. (2023). Transformer neural network to predict and interpret pregnancy loss from activity data in Holstein dairy cows. Comput. Electron. Agric..

[25] Eckhardt R., Arablouei R., Ingham A., McCosker K., Bernhardt H. (2025). Livestock behaviour forecasting via generative artificial intelligence. Smart Agric. Technol..

[26] Kang R., Zhu J., Green S., Brilakis I. (2026). Knowledge graphs for operational decision-making in industrial maintenance: A systematic review. Expert Syst. Appl..

[27] Liu Z., Feng Y., Liu H., Tang R., Yang B., Zhang D., Jia W., Tan J. (2025). TVC Former: A transformer-based long-term multivariate time series forecasting method using time-variable coupling correlation graph. Knowl. Based Syst..

[28] Liu J., Wang X., Xie F., Wu S., Li D. (2023). Condition monitoring of wind turbines with the implementation of spatio-temporal graph neural network. Eng. Appl. Artif. Intell..

[29] Gu Z., Long T., Wang S., Shang X., Shen W., Wei X., Xu K. (2025). Construction of Q&A methods based on knowledge graphs and large language models–improving the accuracy of landscape pest and disease Q&A. Smart Agric. Technol..

[30] Long T., Yu R., You X., Shen W., Wei X., Gu Z. (2025). FSCA-YOLO: An Enhanced YOLO-Based Model for Multi-Target Dairy Cow Behavior Recognition. Animals.

[31] Wan Y., Liu Y., Chen Z., Chen C., Li X., Hu F., Packianather M. (2024). Making knowledge graphs work for smart manufacturing: Research topics, applications and prospects. J. Manuf. Syst..

[32] Wang Y., Peng H., Wang G., Tang X., Wang X., Liu C. (2023). Monitoring industrial control systems via spatio-temporal graph neural networks. Eng. Appl. Artif. Intell..

[33] Jeong G., Jung M., Park S., Park M., Ahn C.R. (2024). Contextual multimodal approach for recognizing concurrent activities of equipment in tunnel construction projects. Autom. Constr..

[34] Lai X., Qiu T., Shui H., Ding D., Ni J. (2023). Predicting future production system bottlenecks with a graph neural network approach. J. Manuf. Syst..

[35] Deng S., Sprangers O., Li M., Schelter S., De Rijke M. (2024). Domain generalization in time series forecasting. ACM Trans. Knowl. Discov. Data.

[36] Liu Y., Huang Z., Zhang F., Zhang X. (2025). A decoupled network with variable graph convolution and temporal external attention for long-term multivariate time series forecasting. Expert Syst. Appl..

[37] Zhang Y., Guinnefollau L., Sullivan M., Phillips C.J.C. (2018). Behaviour and physiology of sheep exposed to ammonia at a similar concentration to those experienced by sheep during export by sea. Appl. Anim. Behav. Sci..

[38] Papanastasiou D.K., Fidaros D., Bartzanas T., Kittas C. (2011). Monitoring particulate matter levels and climate conditions in a Greek sheep and goat livestock building. Environ. Monit. Assess..

[39] Wang Y., Duan Z., Huang Y., Xu H., Feng J., Ren A. (2022). MTHetGNN: A heterogeneous graph embedding framework for multivariate time series forecasting. Pattern Recognit. Lett..

[40] Li Z., Cai R., Chen J., Yan Y., Chen W., Zhang K., Ye J. (2024). Time-series domain adaptation via sparse associative structure alignment: Learning invariance and variance. Neural Netw..

